# The Versatile Photo-Thermal Behaviour of a 2-Hydroxyazobenzene

**DOI:** 10.3390/molecules28031183

**Published:** 2023-01-25

**Authors:** Pier Luigi Gentili, Antonio Capaccioni, Raimondo Germani, Simona Fantacci

**Affiliations:** 1 Department of Chemistry, Biology and Biotechnology, Università degli Studi di Perugia, 06123 Perugia, Italy; 2 Istituto CNR di Scienze e Tecnologie Chimiche “Giulio Natta” (CNR-SCITEC), 06123 Perugia, Italy

**Keywords:** photochromism, photochemistry, molecular probe, molecular switch, azobenzene

## Abstract

Photochromic compounds are employed in implementing neuron surrogates. They will boost the development of neuromorphic engineering in wetware. In this work, the photochromic behaviours of (E)-3,4,6-trichloro-2-(*p*-diazenil)-phenol (***t*-DZH**) and its conjugated phenoxide base (***t*-DZ**) have been investigated experimentally in three different media: (1) pure acetonitrile, (2) in water and acetonitrile mixed in a 1/1 volume ratio, and (3) in an aqueous micellar solution of 3-(*N*,*N*-Dimethylmyristylammonio)propanesulfonate (SB3-14). The analysis of the spectral and kinetic features of ***t*-DZH** and ***t*-DZ** has been supported by quantum-mechanical DFT calculations, the maximum entropy method, and the determination of their colourability (C). The versatility of ***t*-DZH** and ***t*-DZ** makes them promising molecular probes of micro-environments and potential ingredients of photochemical oscillators required for implementing pacemaker neurons capable of communicating through optical signals in wetware.

## 1. Introduction

**Figure 1:**
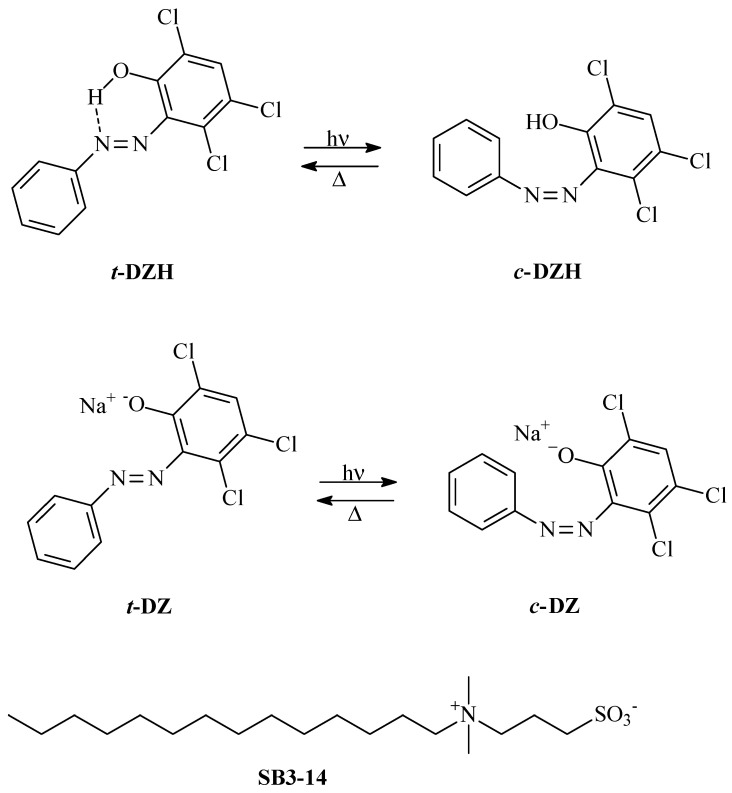
Photochromism of **DZH**, its conjugated base **DZ**, and structure of the **SB3-14** surfactant.

Artificial intelligence (AI) is revolutionizing our societies [1]. It is used in basic and applied science, economy, security, and wellbeing because it supports humans in facing complex scenarios [1,2,3,4]. A promising strategy for developing AI is neuromorphic engineering [1,5]. In neuromorphic engineering, surrogates of neurons are devised through non-biological systems either for neuro-prosthesis [6] or to design brain-like computing machines [5]. Neurons’ surrogates are primarily implemented in hardware or through electrochemical systems [5,7,8,9,10]. However, there exists the possibility of implementing them in wetware [11,12,13,14,15,16]. Surrogates of neurons in wetware are alluring because they can more easily reproduce specific behaviours of real neurons that are intrinsically “wet” and work in a “wet” environment, that of the human brain. Peculiar solutions of nonlinear chemical systems are usually exploited to mimic neural dynamics. They can communicate through chemical or optical signals [15,17]. The most common way for reproducing the dynamics of pacemaker neurons is through oscillatory chemical reactions, such as the well-known Belousov-Zhabotinsky, Briggs-Rauscher, and Orbán reactions [18,19]. The principal drawback of these chemical systems is the need to be open for working indefinitely in time. Everlasting oscillators within closed systems have been recently designed by choosing thermally reversible photochromic compounds as chemical ingredients and a beam of proper UV-visible radiations, having steady intensity, as the power supply [20,21]. It has been experimentally demonstrated that single photochromes can reproduce the dynamics of phasic excitable neurons when electromagnetic radiations are used as signals [22]. The synergy between radiation and convection transforms a solution of a thermally reversible photochromic compound into a dynamical model of a chaotic neuron [23,24]. Finally, a mixture of two peculiar photochromic compounds gives rise to a photochemical oscillator that plays as a dynamic model of a pacemaker neuron [20,21]. Its implementation is difficult because the two photochromes must have specific spectral and kinetic features. More specifically, the two compounds must compete to absorb the excitation radiation, and one of them should react photochemically and thermally much faster than the other. With the intent of finding the right ingredients for the implementation of a photochemical oscillator, the spectral and kinetic properties of the photochromic (E)-3,4,6-trichloro-2-(*p*-diazenil)-phenol (***t*-DZH**) and its conjugated base (***t*-DZ**) (see Figure 1) have been experimentally characterized in three different media: in acetonitrile, in water and acetonitrile mixed in a 1/1 volume ratio, and in an aqueous micellar solution of 3-(*N*,*N*-Dimethylmyristylammonio)propanesulfonate (SB3-14) (Figure 1).

***t*-DZH** is a well-known metastable photoacid [25]. In the E (*trans*) configuration, the acidity of the phenol group is reduced by the formation of a hydrogen bond with a nitrogen atom of the azo group (see Figure 1). The hydrogen bond is broken in the Z (*cis*) isomer. Therefore, the *cis* form is more acidic than the *trans*. ***t*-DZH** has been proposed as a compound to tailor pH pulses and oscillations upon an intermittent UV-irradiation [26,27]. It is also included among the most promising photochemically driven molecular motors [28]. The kinetics and mechanism for the photochromism of hydroxyazobenzenes have been the subject of many theoretical and experimental investigations [29,30]. The presence of a hydrogen bond between the hydroxyl and azo groups favors the formation of a tautomeric hydrazone-like intermediate with a partial breaking of the N=N bond. Hence, the rotation around the N-N bond can occur, facilitating the trans-cis isomerization.

In this work, we quantitatively compare the photochromic features of ***t*-DZH** and its conjugated base ***t*-DZ** in the three distinct media mentioned above. The simple deprotonation of the hydroxyl group significantly transforms the spectral and kinetic properties of the hydroxyazobenzene, as demonstrated in this work through experiments and quantum–mechanical simulations. The versatility of this 2-hydroxyazobenzene makes it a good candidate for the implementation of photochromic oscillators [20,21]. In this work, (E)-3,4,6-trichloro-2-(*p*-diazenil)-phenol and its conjugated base are also evaluated as probes of different micro-environments. Their performances as probes are assessed through cutting-edge algorithms, such as “Fuzzy Entropy” and “Colourability”.

## 2. Results and Discussion

### 2.1. Spectral Features of t-DZH and t-DZ in the Three Media

**Figure 2:**
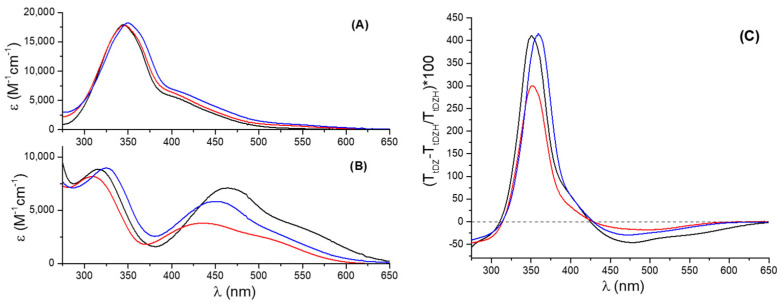
Absorption spectra of ***t*-DZH** in (**A**) and ***t*-DZ** in (**B**) recorded in acetonitrile (black trace), water/acetonitrile = 1/1 (red trace), and in the aqueous micellar solution of SB3-14 (blue trace). Graph (**C**) reports the relative percentage transmittance changes induced by the deprotonation in the three media: acetonitrile (black trace), water/acetonitrile = 1/1 (red trace), and in the aqueous micellar solution of SB3-14 (blue trace).

The absorption spectra of ***t*-DZH** and ***t*-DZ** in the three media are shown in graphs A and B of Figure 2, respectively. They show two principal absorption bands in the spectral range (275–650) nm, as is expected for any azobenzene derivative [31].

**Figure 3:**
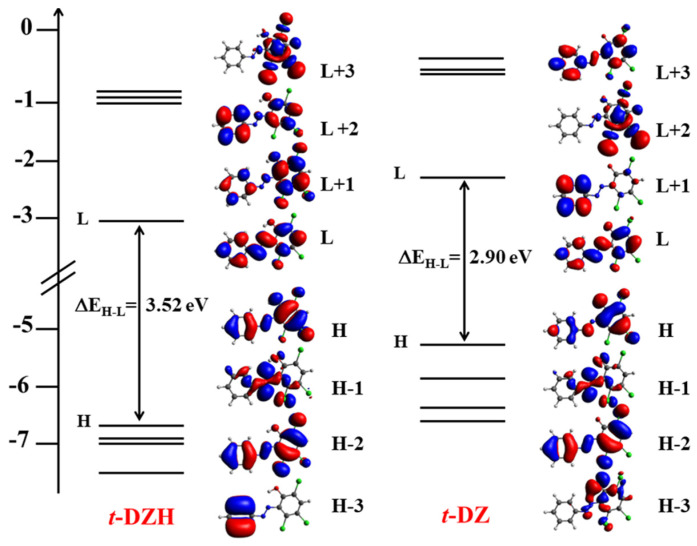
Schematic representation of the energy levels of the frontier molecular orbitals for ***t*-DZH** and ***t*-DZ** along with the isodensity surface plots of HOMO—HOMO−3 and LUMO—LUMO+3 having isodensity contour equal to 0.03 (H and L stand for HOMO and LUMO).

***t*-DZH** has the most intense absorption band peaked at 344 nm in CH_3_CN, 346 nm in H_2_O/CH_3_CN = 1/1, and 350 nm in the aqueous micellar solution of SB3-14 (0.1 M). The absorption coefficient is of the order of $\varepsilon \approx 18,000\;M^{-1}{\mathrm{cm}}^{-1}$, at the maximum of the band. At longer wavelengths, there is a less intense shoulder centred at 425 nm with an absorption coefficient $\varepsilon \approx 4800\;M^{-1}{\mathrm{cm}}^{-1}$. This spectral shoulder has a weak tail that extends up to 600 nm. According to the quantum–mechanical computations (for the methodological details, please read Section 3.6), the most intense band is due to an allowed $\pi \to \pi^{*}$ (S_0_→S_3_) transition involving the HOMO−2→LUMO (91%) and HOMO→LUMO (9%) orbitals as reported in Table 1. The final LUMO orbital has antibonding $\pi^{*}$ character for the N=N group, as shown in Figure 3. Similarly, the shoulder at 425 nm is due to an allowed $\pi \to \pi^{*}$ (S_0_→S_2_) transition involving mainly the HOMO→LUMO (91%) orbitals (see Table 1). The $n\to \pi^{*}$ transition involving the lone pair of the nitrogen atom and the antibonding $\pi^{*}$ orbital of the azo group is shifted towards the red if compared with the $\pi \to \pi^{*}$ transitions. Moreover, it is forbidden and dark according to the quantomechanical calculations (see Table 1). It is responsible for the long weak tail (having $\varepsilon \approx 500\;M^{-1}{\mathrm{cm}}^{-1}$) that extends up to 600 nm.

The deprotonated form ***t*-DZ**, obtained after adding an excess of base (see paragraph 1 of the Supplementary Materials for more details), shows a significantly different spectrum (see Figure 2B). However, it continues to have two principal absorption bands. The band associated with the $\pi \to \pi^{*}$ (HOMO−2→LUMO) transition exhibits hypochromic and hypsochromic effects if compared with the analogous transition detected for ***t*-DZH**: it is peaked at 309 nm in H_2_O/CH_3_CN = 1/1, at 317 nm in CH_3_CN, and at 326 nm in SB3-14, with an average value of its absorption coefficient ($\varepsilon \approx 8700\;M^{-1}{\mathrm{cm}}^{-1}$ estimated on the peak) less than half of the value shown by ***t*-DZH**. The band associated with the $\pi \to \pi^{*}$ (HOMO→LUMO) transition undergoes bathochromic and hyperchromic (excluding ***t*-DZ** in H_2_O/CH_3_CN = 1/1) effects. It is no longer a spectral shoulder, but it is a well-resolved band that is peaked at 435 nm (with $\varepsilon =3792\;M^{-1}{\mathrm{cm}}^{-1}$) in H_2_O/CH_3_CN = 1/1, 451 nm (with $\varepsilon =5822\;M^{-1}{\mathrm{cm}}^{-1})$ in SB3-14, and 463 nm (with $\varepsilon =7095\;M^{-1}{\mathrm{cm}}^{-1})$ in CH_3_CN. The smallest value of the absorption coefficient is in the mixed solvent H_2_O/CH_3_CN = 1/1. It is probably due to the possibility of establishing hydrogen bonds between the azo group and the water molecules.

Graph C of Figure 2 reports the relative percentage transmittance (T) changes induced by the deprotonation of ***t*-DZH**, defined as:

(1) $$\left(\frac{T_{tDZ}-T_{tDZH}}{T_{tDZH}}\right)100$$

The addition of an excess of a strong base to a solution of ***t*-DZH** $5\times 10^{-5}\;M$ in the three media determines transmittance decreases between 425 and 650 nm and below 311 nm, and transmittance increases between 311 and 425 nm. The transmittance spectra of ***t*-DZH** and ***t*-DZ**, having concentrations of $5\times 10^{-5}\;M$ and reported in paragraph 3 of the Supplementary Materials are transformed in chromaticity coordinates x, y, and z according to the procedure described in Materials and Methods Section 3.5. The results are reported in Table 2 (For the XYZ tristimulus and the RGB values, please look at Table S1). It is possible to quantify the colour changes induced by the deprotonation through the determination of the colourability (C), which is the difference between the information sent by ***t*-DZ (**$I_{tDZ})$ and that sent by ***t*-DZH (**$I_{tDZH})$, according to Shannon’s theory of information [32,33]:

(2) $$\begin{array}{cc} C=I_{tDZ}-I_{tDZH} & =x_{tDZ}\log_{2}(x_{tDZ})-x_{tDZH}\log_{2}(x_{tDZH})+y_{tDZ}\log_{2}(y_{tDZ})\\ & -y_{tDZH}\log_{2}(y_{tDZH})+z_{tDZ}\log_{2}(z_{tDZ})-z_{tDZH}\log_{2}(z_{tDZH}) \end{array}$$

In Equation (2), C is the difference between the information that ***t*-DZ** and ***t*-DZH** send to a human eye in case 2-hydroxyazobenzene is used as a sensor. Such information is expressed through the well-known Shannon’s formula and is based on the colour coordinates values reported in Table 2. It results (see the last column of Table 2) that CH_3_CN assures the most considerable C value, whereas the mixture H_2_O/CH_3_CN = 1/1 the smallest one.

### 2.2. Photochromism of t-DZH in the Three Media

**Figure 4:**
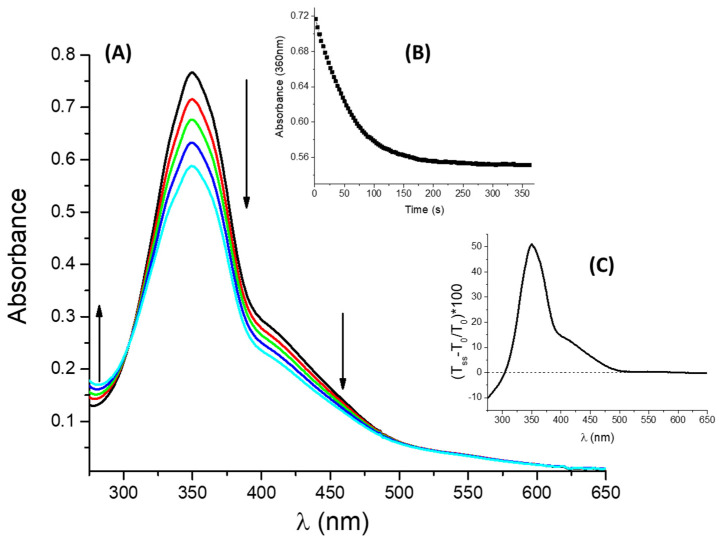
Spectral modifications (**A**) induced by a stationary UV irradiation at $\lambda_{irr}=\left(363\pm 40\right)\;\mathrm{nm}$ for [***t*-DZH**] = $4.2\times 10^{-5}\;M$ in [SB3-14] = 0.1 M: the arrows indicate the trends of absorbance in the different spectral regions (the black and cyan traces are the initial and final spectra, recorded before irradiation and at the photo-stationary state, respectively). Graph (**B**) shows how the absorbance at 360 nm changes upon UV irradiation. Graph (**C**) reports the trend of the relative percentage transmittance change, defined in Equation (3), as the function of wavelength.

***t*-DZH** was irradiated by a UV band with wavelengths included in the range $\lambda_{irr}=\left(363\pm 40\right)\mathrm{nm}$ (see paragraph 3.3 for more details). The irradiation of ***t*-DZH** determines the trans-to-cis photo-isomerization. The spectral modifications recorded upon stationary UV irradiation and in a micellar solution of SB3-14 (0.1 M) are shown in Figure 4A. The spectral evolution reveals an isosbestic point at 304 nm. For λ>304 nm, the absorbance decreases, whereas for the wavelengths included in (275–303) nm the absorbance slightly increases. The trend of the absorbance at 360 nm (A(360 nm)) recorded upon stationary irradiation is reported in Graph B of Figure 4. A(360 nm) decreases monotonically until a plateau is reached. The plateau value corresponds to the photo-stationary state condition. Figure 4C shows the relative percentage of transmittance changes induced by the photochemical *trans*-to-*cis* reaction and is defined as:

(3) $$\left(\frac{T_{ss}-T_{0}}{T_{0}}\right)100$$

wherein $T_{0}$ and $T_{ss}$ are the initial and final (at the photo-stationary state) transmittance values, respectively. The transmittance change profile reproduces the original spectral shape of ***t*-DZH**. Therefore, it is reasonable to infer that the spectral profile for the *cis* is qualitatively similar to that for ***t*-DZH**. It is characterized by smaller absorption coefficient values for λ>304 nm and larger ones for λ∈ (275–303) nm. At 304 nm, $\varepsilon_{cis}=\varepsilon_{tDZH}$. The decrease in absorbance in the visible region is responsible for a negative value of the colourability defined as $C=I_{ss}-I_{0}$ (wherein $I_{ss}$ and $I_{0}$ are the information sent by the chemical system to naked human eyes at the steady-state and before irradiation, respectively). For the spectra reported in Figure 4 and recorded in an aqueous micellar solution with [SB3-14] = 0.1 M and [***t*-DZH**] = $4.2\times 10^{-5}\;M$, $C=-2.3\times 10^{-3}$ (see Table S2 for the colour coordinates values used for the calculation of C).

The spectral evolutions recorded under stationary UV irradiation for ***t*-DZH** dissolved in H_2_O/CH_3_CN = 1/1 and acetonitrile are shown in Figures S10 and S11, respectively. They show an isosbestic point at 300 and 297.5 nm, respectively. The associated changes in the values of the colour coordinates and the colourability C values are reported in Tables S3 and S4, respectively. Only in CH_3_CN, it is possible to detect a slight increase in absorbance in the visible region, between 500 and 600 nm, upon UV irradiation. It is due to the expected growth of the absorption coefficient for the $n\to \pi^{*}$ transition passing from the trans to the cis isomer [31]. An effect of this peculiar behaviour is that the colourability C is positive, but tiny, just in acetonitrile. The values of the photochemical quantum yield $\Phi_{t\to c}$ for ***t*-DZH** dissolved in the three media were determined according to the procedure explained in Section 3.3 of the Materials and Methods section. The final results are reported in Table 3. $\Phi_{t\to c}$ is almost the same in the binary mixture H_2_O/CH_3_CN = 1/1 and the micellar solution of SB3-14, whereas it is more than twice larger in acetonitrile.

**Figure 5:**
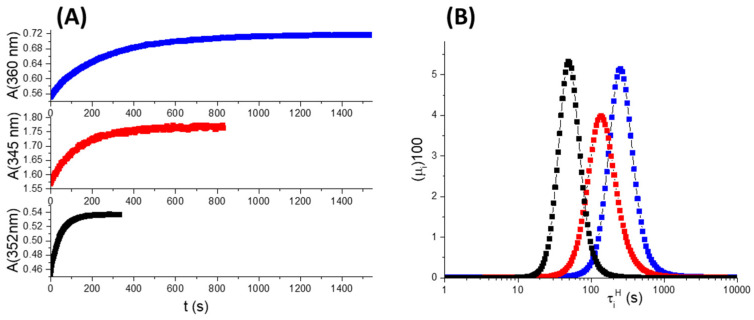
Graph (**A**): Kinetics of the thermal *cis*-to-*trans* isomerization for **DZH** in the aqueous micellar solution of SB3-14 0.1 M (blue trace); in H_2_O/CH_3_CN = 1/1 (red trace); in CH_3_CN (black trace). Graph (**B**): lifetimes distributions of the ***c*-DZH** conformers, determined through the maximum entropy method.

The three media significantly affect the thermal *cis*-to-*trans* isomerization, as shown by the kinetics plotted in Figure 5A. The isomerization when ***c*-DZH** is enclosed within the SB3-14 micelles requires about 1000 s to be complete (see the blue trace); in H_2_O/CH_3_CN = 1/1, it takes about 600 s (red trace), and in pure CH_3_CN, just 100 s or so (black trace). The thermal kinetics were fitted using the least-squares (LS) method applied to the mono-exponential function shown in Equation (11), and the maximum entropy method applied to the poly-exponential function of the type shown in Equation (12). More details about the maximum entropy method (MEM) can be found in Section 3.4 of the Materials and Methods section. The results of MEM analysis are plotted in Figure 5B, whereas those of LS method are reported in Table 4. Graph 5B shows the lifetimes of the *cis* isomers ($\tau_{i}^{H}$) expressed in seconds, and their percentage weights ($\mu_{i}\times 100$). The black band refers to the lifetimes’ distribution for ***c*-DZH** in CH_3_CN. It is peaked at 49 s, with $\mu_{i}\times 100=5.3\%$. The lifetime that corresponds to the peak is in agreement with the output of the LS method that gave $\tau_{LS}^{H}=50\;s$. The degree of micro-heterogeneity can be quantitatively estimated by calculating the normalized fuzzy entropy, according to the following equation [34]:

(4) $$H_{nor}=-\frac{1}{log\left(N\right)}\overset{N}{\underset{i=1}{\sum }}\mu_{i}log\left(\mu_{i}\right)$$

In (4), N is the number of exponential terms. $H_{nor}$ assumes values included between 0 and 1. The larger the micro-heterogeneity, the bigger the $H_{nor}$ value [35]. The $H_{nor}$ value for ***c*-DZH** in acetonitrile is 0.62, as reported in Table 4. The micro-heterogeneity in the pure acetonitrile can be ascribed almost exclusively to the distribution of conformers for ***c*-DZH**. The DFT calculations revealed that ***c*-DZH** exists in many, almost iso-energetic, conformers (for more details, please read paragraph 5 of the Supplementary Materials).

The red band appearing in Figure 5B refers to the lifetimes’ distribution for ***c*-DZH** in H_2_O/CH_3_CN = 1/1. It is peaked at 136 s (in agreement with the output of the LS method that gave $\tau_{LS}^{H}=$137 s), with $\mu_{i}\times 100=4.0\%$. The presence of water slows down the isomerization rate, probably due to the hydrogen bonds that can be established between H_2_O molecules and the polar groups of ***c*-DZH**. The mixed solvent originates a higher degree of micro-heterogeneity, as confirmed by the larger $H_{nor}$ value, which is 0.69 (see Table 4).

Finally, the blue band of Figure 5B refers to the lifetimes’ distribution for ***c*-DZH** in the micellar solution of SB3-14. It is peaked at 252 s (in good agreement with the output of the LS method that gave $\tau_{LS}^{H}=$ 259 s), with $\mu_{i}\times 100=5.1\%$. The confined micro-environment of the micelles provokes a further decrease in the *cis*-to-*trans* isomerization rate. The “braking” effect of the zwitterionic SB3-14 micelles is significantly more relevant than that exerted by other micelles constituted by neutral (for instance, octyl-β-D-glucopyranoside) and cationic (for instance, cetyltrimethylammonium chloride) surfactants [27]. According to the value of $H_{nor}=0.67$, the degree of micro-heterogeneity for ***c*-DZH** within the SB3-14 micelles (***c*-DZH** cannot be in the bulky solvent due to its tested insolubility in water) is in between that estimated in pure acetonitrile and that in H_2_O/CH_3_CN = 1/1 mixture.

### 2.3. Photochromism of t-DZ in the Three Media

**Figure 6:**
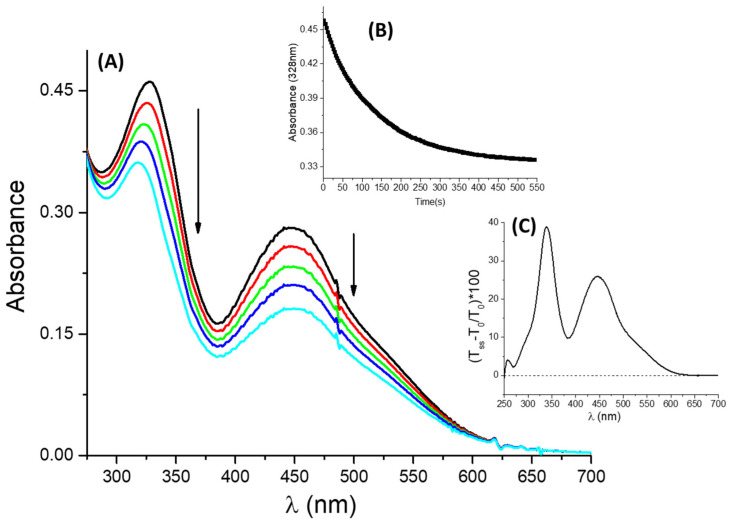
Spectral modifications (**A**) induced by a stationary UV irradiation at $\lambda_{irr}=\left(363\pm 40\right)\;\mathrm{nm}$ for [***t*-DZ**] = $4.8\times 10^{-5}\;M$ in [SB3-14] = 0.1 M: the arrows indicate the trends of absorbance in the different spectral regions (the black and cyan traces are the initial and final spectra, recorded before irradiation and at the photo-stationary state, respectively). Graph (**B**) shows how the absorbance at 328 nm changes upon UV irradiation. Graph (**C**) reports the trend of the relative percentage transmittance change, defined in Equation (3), as the function of wavelength.

***t*-DZ,** obtained by deprotonation of its conjugated acid through the injection of an excess of a strong base (as explained in Section 2.1), was irradiated by a UV band with $\lambda_{irr}=\left(363\pm 40\right)\mathrm{nm}$ (see Section 3.3 for more details). The irradiation of ***t*-DZ** determines the *trans*-to-*cis* photo-isomerization. The spectral modifications recorded upon stationary UV irradiation and in a micellar solution of SB3-14 (0.1 M) are shown in Figure 6A. The spectral evolution reveals a decrease in absorbance, i.e., an increase in transmittance (see Figure 6C), extended to the entire region between 250 and 620 nm. Graph 6B reports the trend of the absorbance at 328 nm as the function of the steady irradiation time. The decrease in absorbance in the visible region is responsible for a negative value of the colourability C, as defined in Equation (2). For the spectra reported in Figure 6 and recorded in an aqueous micellar solution with [SB3-14] = 0.1 M and [***t*-DZH**] = $4.8\times 10^{-5}\;M$, $C=-9.8\times 10^{-3}$ (the values of the colour coordinates used for C determination are reported in Table S5).

The spectral evolutions of the ***t*-DZ** solutions under UV irradiation in the H_2_O/CH_3_CN = 1/1 mixture and pure CH_3_CN are similar to that reported in Figure 6. They are shown in Figures S12 and S13. The absorbance decreases, whereas the transmittance increases in the entire range, between 250 and 650 nm, due to the *trans*-to-*cis* photo-isomerization. Of course, the colourability C, estimated considering the two spectra recorded at the photo-stationary state and before irradiation, respectively, results to be negative, as reported in Tables S6 and S7. Following the procedure described in Section 3.3 of the Materials and Methods, the values of the photochemical quantum yield $\Phi_{t\to c}$ for ***t*-DZ** dissolved in the three media were determined. The results are listed in Table 5. The binary mixture H_2_O/CH_3_CN = 1/1 guarantees the largest $\Phi_{t\to c}$. The value in pure acetonitrile is comparable to that determined for ***t*-DZH** in the same solvent, although slightly larger. When ***t*-DZ** is confined in the SB3-14 micellar environment, it shows the lowest $\Phi_{t\to c}$. This evidence agrees with what has been found for ***t*-DZH** (see Table 3). In any way, the $\Phi_{t\to c}$ values for ***t*-DZ** are larger than those for ***t*-DZH** if compared in the same medium (compare the $\Phi_{t\to c}$ values reported in Table 5 with those in Table 3). The absence of the proton from the hydroxyl group makes the photochemical process more probable.

**Figure 7:**
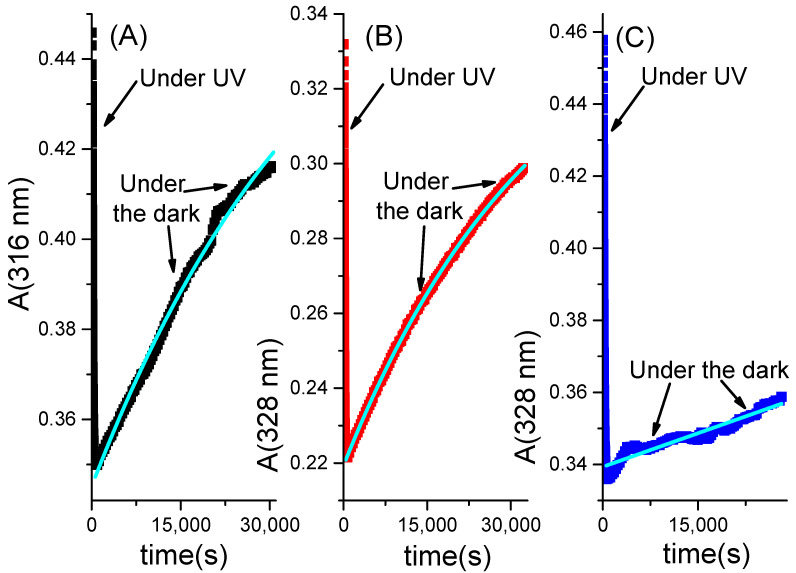
Trends of the absorbance at one specific wavelength for **DZ** that photo-isomerizes from *trans*-to-*cis* in the portion of absorbance that decays and isomerizes thermally from *cis*-to-*trans* in the portion of absorbance that grows. Graphs (**A**–**C**) refer to **DZ** in CH_3_CN, H_2_O/CH_3_CN = 1/1, and aqueous micellar solution of [SB3-14] = 0.1 M, respectively. The cyan curves are the functions fitting only the growth that corresponds to the thermal isomerization.

The absence of the proton has the opposite effect on the thermal *cis*-to-*trans* isomerization reaction. Figure 7 shows the evolution of the absorbance at specific wavelengths for **DZ** that is, at first, under UV irradiation and, then, under the dark. Under UV irradiation, the absorbance decreases quickly, as already shown in Figure 6, Figures S12 and S13. After reaching the photo-stationary state, the UV irradiation is discontinued, and a prolonged thermal *cis*-to-*trans* reaction occurs. The latter reaction generates a slow increase in the absorbance, as shown in graphs 7A, 7B, and 7C for ***c*-DZ** dissolved in CH_3_CN, H_2_O/CH_3_CN = 1/1, and a micellar solution of SB3-14, respectively. The trends of absorbance under the dark reveal that the *cis*-to-*trans* thermal reactions are not complete within about 30,000 s (i.e., roughly 8 h and 20 min). The cyan traces appearing in the three graphs are the outputs of the fitting procedures applied to estimate the lifetimes of the cis isomer in the three media, based on the data collected in the first 8 and ½ h of the thermal *cis*-to-*trans* reaction. The fitting function is that shown in Equation (11). The best estimates of the lifetimes, determined using the least-squares method, are reported in Table 6. The mixture H_2_O/CH_3_CN = 1/1 guarantees the shortest lifetime for ***c*-DZ** among the three media: it lasts almost 10 h. In CH_3_CN, it is about 1 h and a half longer. In the micellar solution of SB3-14, it is incredibly much longer. In the first 8 h, the growth is so tiny that it looks linear and can be fitted by the function reported in Equation (5), which has been obtained as the McLaurin series expansion of the original fitting function (11):

(5) $$A^{\lambda_{\mathrm{an}}}\approx {\left(A^{\lambda_{\mathrm{an}}}\right)}_{\mathrm{ss}}+\left({\left(A^{\lambda_{\mathrm{an}}}\right)}_{0}-{\left(A^{\lambda_{\mathrm{an}}}\right)}_{\mathrm{ss}}\right)\left(k_{\Delta }t\right)$$

The slope of the linear function (5), determined through the least-squares method, allowed to infer that $\tau_{LS}$ is about 22 days and 17 h. The SB3-14 micelles exert a remarkable “braking” effect on the thermal *cis*-to-*trans* isomerization. It is well known that when *cis*-azobenzene and its derivatives isomerize thermally, they might not be strongly influenced by steric hindrances [31,36,37,38]. Therefore, the relevant “braking” effect exerted by SB3-14 micelles might be mainly due to electrostatic interactions that can be established between the negatively charged ***c*-DZ** molecule and the positive charge localized in the nitrogen atom of the zwitterionic SB3-14 molecule.

Finally, it is worthwhile noticing that the proton of the hydroxylic group in **DZH** is a formidable catalyst of *cis*-to-*trans* isomerization. The intramolecular hydrogen bond that establishes between the hydroxylic and the azo groups is of the order of 4.73 Kcal/mol according to the DFT simulations (see Figure S15). If we compare the cis lifetimes reported in Table 4 and Table 6, we deduce that H^+^ speeds up the thermal isomerization 259 times in H_2_O/CH_3_CN = 1/1, 820 times in CH_3_CN, and even 7600 times in SB3-14 micelles. The formation of a hydrogen bond in the *trans* isomer gives rise to a six-membered ring that significantly stabilizes the *trans* isomer and the transition state that is formed when the *cis* transforms into the *trans* isomer. Furthermore, the proton can catalyze the *cis*-to-*trans* conversion through the formation of the tautomeric phenylhydrazone [39].

### 2.4. Photo-Induced ΔpH

**Figure 8:**
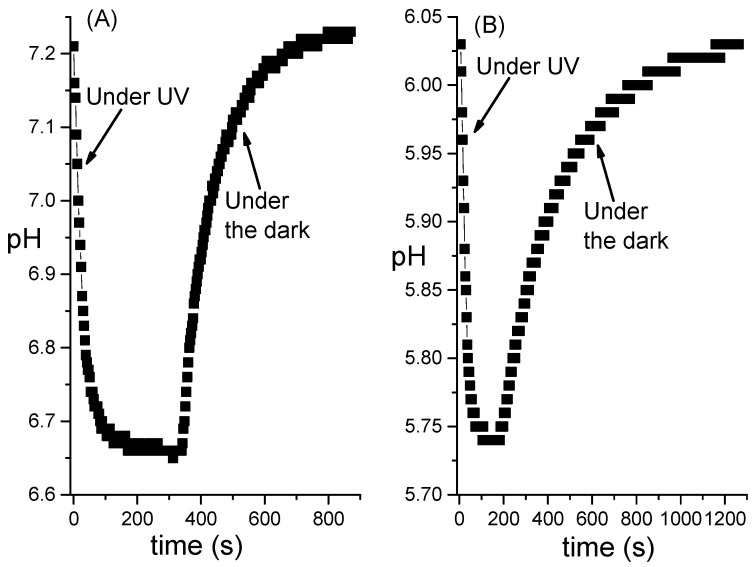
pH changes generated by [***t*-DZH**] = $1\times 10^{-4}M$ dissolved in H_2_O/CH_3_CN = 1/1 (**A**) and in an aqueous micellar solution of SB3-14 0.1 M (**B**). The pH drops were photo-induced using the same irradiation source (see Figure S16 and the end of Section 3.3 for more details).

The results presented in the previous paragraphs reveal that 2-hydroxyazobenzene is a truly versatile photochromic compound. Its behaviour is sensitive to the features of the medium in which is embedded and changes noticeably going from **DZH** to **DZ** after its deprotonation. ***t*-DZH** has another well known property that makes it promising in the field of neuromorphic engineering: it is its photoacid behaviour. Under UV irradiation, it photo-isomerizes, and the product, i.e., ***c*-DZH**, is a stronger acid than ***t*-DZH**. Therefore, it is possible to photo-induce a pH drop, as shown in Figure 8. Graph A refers to H_2_O/CH_3_CN = 1/1 solution and ΔpH=−0.55. Graph B refers to an aqueous micellar solution of [SB3-14] = 0.1 M, irradiated with the same lamp and at the same intensity (see the end of Section 3.3 for more details), but originating a ΔpH=−0.29. The smaller ΔpH is presumably due to the buffer role played by the SB3-14 micelles, which also shift the absolute pHs to more acidic values.

Figure 8 shows that the pH’s change is reversible: by maintaining the solutions under the dark, a complete recovery of the original pHs were observed.

The photo-acid behaviour makes ***t*-DZH** capable of communicating with other compounds chemically through the release of a proton. This property extends the possibility of coupling a pair of photochromic compounds that can interplay through light and protons. They might allow the implementation of the first photochemical oscillator as a dynamical model of a pacemaker neuron. Anyway, a solution of the simple ***t*-DZH** can be exploited to mimic the dynamics of neurons in the phasic excitable regime, which respond to external stimuli in an analog manner [24,40]. The extents of ΔpH (shown in Figure 8) are proportional to the irradiation intensity when the concentration of ***t*-DZH** has been fixed. As soon as the irradiation is discontinued, the compound recovers the original state spontaneously and reversibly, as any phasic excitable neuron does. The reversibility is shown in Figure S17.

## 3. Materials and Methods

### 3.1. Materials

Milli Q water (18 MΩ, pH 6.50) and acetonitrile from Sigma-Aldrich, distilled over P_2_O_5_ before use, were employed as solvents. The commercial grade zwitterionic surfactant 3-(*N*,*N*-Dimethylmyristylammonio)propanesulfonate (SB3-14) was purchased from Fluka and was crystallized twice from an acetone–methanol mixture before using [41]. The value of the critical micelle concentration (CMC = 2.88 × 10^−4^ M) was determined from plots of surface tension vs. −log[surfactant]. No minima could be observed in these plots. Surface tensions were measured on a Fischer, du Nouy type tensiometer at room temperature. In this work, micellar solutions with [SB3-14] = 0.1 M were prepared. The strong bases 1,8-diazabicyclo[5.4.0]undec-7-ene (DBU) and NaOH, purchased from Sigma-Aldrich (St. Louis, MO, USA), were used without further purification. The photochromic compound 6-morpholino-3-(4-morpholinophenyl)-3-phenyl-3H-naphtho[2,1-b]pyran (PHT) synthesized by M. Heron’s group (Huddersfield University, UK) [42] was used as the actinometer.

### 3.2. Synthesis of t-DZH

***t*-DZH** was synthesized in agreement with the literature procedures [26,43,44,45]. A solution of sodium nitrite (1.656 g, 6 mmol) in water (20 mL) cooled to 3–5 °C was added in small volumes to a continuously stirred solution of freshly distilled aniline (0.986 g, 5.4 mmol) in HCl conc. (4 mL) and water (4 mL) maintained at 3–5 °C. The resulting diazonium salt solution was then added very slowly to a solution of 2,4,5-trichlorophenol (2.132 g, 5.4 mmol, purchased from Carlo Erba and purified by sublimation) in 10% (*w*/*v*) NaOH (30 mL) maintained at 3–5 °C. At the end of the additions, the solution was left for 1 h under stirring in an ice-water bath and, finally, acidified with HCl (conc.) until complete precipitation of the diazo compound as a brick-red solid. The resulting solid was filtered on a Buchner, washed with cold water until neutral, crystallized in a water/ethanol mixture (1/1), and vacuum dried over P_2_O_5_. Yield: 76%. Melting point (determined on the Barloworld Scientific Stewart SMP3 apparatus) = 128–130 °C. ^1^H-NMR spectrum (recorded on a Bruker Avance III HD 400 MHz instrument using CDCl_3_ as solvents at 25.0 °C) showed the following chemical shifts (δ) in ppm relative to the residual ^1^H solvent signal: 7.94 (m, 3H); 7.62 (m, 2H); 7,22 (m, 1H).

### 3.3. Spectrophotometric and Photochemical Experiments

The absorption spectra were recorded by a Hewlett-Packard 8453 diode array spectrophotometer. The radiation emitted by a 150 W Xe lamp was filtered by the short pass filter UG1 (Horiba Jobin Yvon), whose transmittance spectrum is shown in Figure S8. The transmitted UV radiation was focused on a 1 cm path length fluorimetric quartz cuvette, perpendicular to the monitoring beam of the spectrophotometer through a 0.6 cm diameter silica optical fiber. It was exploited as the irradiation source. Its intensity was determined by using 6-morpholino-3-(4-morpholinophenyl)-3-phenyl-3H-naphtho[2,1-b]pyran (PHT) as the actinometer because its quantum yield of photocolouration was previously determined [42]. The quantum yield of *trans*→*cis* photoisomerization has been determined using the method of the initial rates. The time variation of the *trans* concentration irradiated at $\lambda_{irr}$ can be expressed as:

(6) $$\frac{d\left[\mathrm{trans}\right]}{\mathrm{dt}}=-\Phi_{t\to c}I_{\mathrm{abs}}^{\mathrm{trans}}+k_{\Delta }\left[\mathrm{cis}\right]\;$$

In Equation (6), $\Phi_{t\to c}$ is the quantum yield, $I_{abs}^{trans}$ is the intensity absorbed by the *trans* isomer and $k_{\Delta }$ is the kinetic constant of the thermal *cis*→*trans* isomerization. The contribution of the photochemical conversion of *cis*→*trans* is neglected because the absorption coefficient and concentration of the *cis* isomer are significantly smaller than those of the *trans* isomer in the initial part of the irradiation. $I_{abs}^{trans}$ can be expressed by the following equation:

(7) $$I_{\mathrm{abs}}^{\mathrm{trans}}=\frac{A_{\mathrm{trans}}^{\lambda_{\mathrm{irr}}}}{A_{\mathrm{tot}}^{\lambda_{\mathrm{irr}}}}I_{0}\left(1-10^{-A_{\mathrm{tot}}^{\lambda_{\mathrm{irr}}}}\right)=A_{\mathrm{trans}}^{\lambda_{\mathrm{irr}}}I_{0}F\;$$

where in $A_{trans}^{\lambda_{irr}}$ and $A_{tot}^{\lambda_{irr}}$ are the absorbances of *trans* and *trans*+*cis* at $\lambda_{irr}$, respectively. $I_{0}$ is the intensity emitted by the lamp at $\lambda_{irr}$. $F=\left(1-10^{-A_{tot}^{\lambda_{irr}}}\right)/A_{tot}^{\lambda_{irr}}$ is known as the photokinetic factor. Introducing Equation (7) into (6) and substituting $\left[cis\right]=(C_{0}-\left[trans\right])$ with $C_{0}$ being the analytical concentration of ***t*-DZH**, the original differential equation assumes the following form:

(8) $$\frac{d\left[\mathrm{trans}\right]}{\mathrm{dt}}=-\Phi_{t\to c}A_{\mathrm{trans}}^{\lambda_{\mathrm{irr}}}I_{0}F+k_{\Delta }\left(C_{0}-\left[\mathrm{trans}\right]\right)\;$$

If we multiply both terms of Equation (8) by $\varepsilon_{trans}^{\lambda_{an}}l$, where $\varepsilon_{trans}^{\lambda_{an}}$ is the absorption coefficient of *trans* at the wavelength of analysis (which is different from $\lambda_{irr}$) and l is the optical path length, then Equation (9) is achieved:

(9) $$\frac{{\mathrm{dA}}_{\mathrm{trans}}^{\lambda_{\mathrm{an}}}}{\mathrm{dt}}=k_{\Delta }\varepsilon_{\mathrm{trans}}^{\lambda_{\mathrm{an}}}{\mathrm{lC}}_{0}-\left(k_{\Delta }+\Phi_{t\to c}\varepsilon_{\mathrm{trans}}^{\lambda_{\mathrm{irr}}}I_{0}\mathrm{Fl}\right)A_{\mathrm{trans}}^{\lambda_{\mathrm{an}}}\;$$

The values of $\Phi_{t\to c}$ for both ***t*-DZH** and ***t*-DZ** in the three media (CH_3_CN, H_2_O:CH_3_CN = 1:1, SB3-14) were determined using the initial rates method. In the first ten seconds of irradiation, Equation (9), showing a linear relationship between the derivative $\left(dA_{trans}^{\lambda_{an}}/dt\right)$ and $A_{trans}^{\lambda_{an}}$, was applied. The slope of the linear relationship, determined through the least-squares method, is:

(10) $$b=\left(k_{\Delta }+\Phi_{t\to c}\varepsilon_{\mathrm{trans}}^{\lambda_{\mathrm{irr}}}I_{0}\mathrm{Fl}\right)\;$$

The photokinetic factor F (see Equation (7)) was calculated considering the average value of $A_{tot}^{\lambda_{irr}}$ in the first ten seconds of irradiation. $k_{\Delta }$ was calculated by fitting the time evolution of $A^{\lambda_{an}}$ after reaching the stationary state and discontinuing the irradiation through the following exponential function:

(11) $$A^{\lambda_{\mathrm{an}}}={\left(A^{\lambda_{\mathrm{an}}}\right)}_{0}-\left({\left(A^{\lambda_{\mathrm{an}}}\right)}_{0}-{\left(A^{\lambda_{\mathrm{an}}}\right)}_{\mathrm{ss}}\right)e^{-k_{\Delta }\left(t\right)}\;$$

wherein ${\left(A^{\lambda_{an}}\right)}_{0}$ and ${\left(A^{\lambda_{an}}\right)}_{ss}$ are the absorbance values at $\lambda_{an}$ determined before irradiation and at the photo-stationary state. The best estimates of the parameters appearing in Equation (11) have been obtained using the least-squares method.

The intensity $I_{0}$ was determined using the thermally reversible photochromic compound PHT as the actinometer. The absorption spectra of the uncoloured (Un) and coloured (Co) forms are shown in Figure S9. Equations (6)–(10) were applied to PHT after substituting *trans* with Un and *cis* with Co. From the slope b defined in (10), it was possible to evaluate $I_{0}$ being $\Phi_{Un\to Co}=0.24$ at $\lambda_{irr}=363\;\mathrm{nm}$ according to the results reported in reference [42].

The data shown in Figure 8 were collected by using a Sension+ MM374 GLP multimeter (HACH) immersed in 40 mL solution of ***t*-DZH** (contained in a Becker) under continuous stirring and thermostated at 293 K. The spectral profile and the quantitative radiance of the 300 W lamp are reported in Figure S16. The irradiation was carried out from above on a surface area of 20 cm^2^.

### 3.4. Determination of the c-DZH Lifetimes through the Maximum Entropy Method

The spectrophotometric thermal isomerization of the *cis*-to-*trans* isomers for **DZH** in the three media have been analyzed through the maximum entropy method (MEM) using the MemExp Software [46]. The fitting function, derived from Equation (11)*,* has the following form:

(12) $$A^{\lambda_{\mathrm{an}}}={\left(A^{\lambda_{\mathrm{an}}}\right)}_{0}-\overset{N}{\underset{i=1}{\sum }}\Delta A_{i}e^{-\frac{t}{\tau_{i}}}\;$$

It is MEM that determines the least number of exponential terms needed to describe the experimental spectrophotometric kinetics by maximizing the function Q:

(13) $$Q=S-{\lambda \chi }^{2}-\alpha I\;$$

In (13), $S=-\sum_{i=1}^{N\to \infty }\mu_{i}log\left(\mu_{i}\right)$ is Shannon’s entropy with $\mu_{i}=\left(\Delta A_{i}/\sum_{i=1}^{N}\Delta A_{i}\right)$; I is a normalization factor, and λ and α are Lagrange multipliers. In the definition of $\chi^{2}$, the standard errors in the experimental data were assumed to be Gaussian type. The output of MEM gives the relative weights of the lifetimes $\tau_{i}$ for i=1,…, N. The output of MEM has been demonstrated to be powerful for retrieving information about the micro-heterogeneity of diverse chemical samples [34,47,48].

### 3.5. Method for Calculating the Chromaticity and RGB Coordinates from the Transmittance Spectra

The transmittance spectra are transformed in chromaticity coordinates *x*, *y*, *z* by the following procedure [33,49]. First of all, the CIE XYZ tristimulus values have been calculated through the integrals:

(14) $$\begin{array}{c} X=\frac{1}{k}\int_{360}^{800}D\left(\lambda \right)T\left(\lambda \right)\overset{-}{x}\left(\lambda \right)d\lambda \\ Y=\frac{1}{k}\int_{360}^{800}D\left(\lambda \right)T\left(\lambda \right)\overset{-}{y}\left(\lambda \right)d\lambda \\ Z=\frac{1}{k}\int_{360}^{800}D\left(\lambda \right)T\left(\lambda \right)\overset{-}{z}\left(\lambda \right)d\lambda \end{array}$$

where in $\overset{-}{x},\overset{-}{y},\overset{-}{z}$ are the colour-matching functions whereby the CIE (Commision Internationale de l’Éclairage) standardized the sensitivity of human eye in 1964; *D*(*λ*) is the energy distribution of the CIE normalized illuminant D65 (which closely matches that of the sky daylight); *T*(*λ*) is the transmittance spectrum, and k is a normalization factor defined in such a way that a sample with a uniform transmittance *T*(*λ*) = 1 for *λ* ∈ (360–800) gives a luminance component *Y* = 1:

(15) $$k=\int_{360}^{800}D\left(\lambda \right)\overset{-}{y}\left(\lambda \right)d\lambda$$

Then, the chromaticity coordinates are calculated as follows:

(16) $$\begin{array}{c} x=X/\left(X+Y+Z\right)\\ y=Y/\left(X+Y+Z\right)\\ z=Z/\left(X+Y+Z\right) \end{array}$$

From Equation (16), it derives that *x* + *y* + *z* = 1.

The *XYZ* tristimulus values have been transformed into the RGB coordinates by the following linear transformation:

(17) $$\left[\begin{array}{c} R\\ G\\ B \end{array}\right]=\left[\begin{array}{ccc} 3.240479 & -1.537150 & -0.498535\\ -0.969256 & 1.875992 & 0.041556\\ 0.055648 & -0.204043 & 1.057311 \end{array}\right]\left[\begin{array}{c} X\\ Y\\ Z \end{array}\right]$$

The RGB values should stay between 0 and 1. When they were slightly greater than 1, their values were rounded to 1. The final step was to scale the RGB values obtained from (17) to values included between 0 and 255.

### 3.6. Quantum-Mechanical Simulations

All the quantum-mechanical calculations performed to unveil some spectral and kinetic features of the photochromic 3,4,6-trichloro-2-(*p*-diazenil)-phenol are based on the density functional theory (DFT) [50] applied through Gaussian09 (G09) software, without any symmetry constraints [51]. The molecular geometries of ***t*-DZH**, ***c*-DZH**, ***t*-DZ** and ***c*-DZ** were investigated using B3LYP [52,53,54] as the exchange–correlation functional and 6-311++g** as the basis set [55,56]. The molecular structures were optimized in acetonitrile solution, including solvation effects through the conductor-like polarizable continuum model (C-PCM) as implemented in G09 [57,58,59]. The dispersion effects by means of the D3-BJ model were included in the molecular geometry optimization of the *cis* isomers [60,61]. Time-dependent DFT (TDDFT) calculations at the B3LYP/6-311++G**/CPCM level of theory were carried out on the optimized geometries of ***t*-DZH** and ***t*-DZ** to simulate their absorption spectra and assign the spectral bands to specific electronic transitions. The non-equilibrium version of the C-PCM was employed for TDDFT calculations, as implemented in G09 [62,63]. To simulate the optical spectra, the 20 lowest spin-allowed singlet–singlet transitions were computed starting from the most stable conformation of the electronic ground state. Transition energies and oscillator strengths were interpolated by a Gaussian convolution with a σ value of 0.15 eV, and maps of density differences between the lowest excited states and the ground states were built to visualize the charge transfers associated with the electronic transitions.

## 4. Conclusions

The data collected and processed in this work outline some relevant photochromic properties of (E)-3,4,6-trichloro-2-(*p*-diazenil)-phenol (***t*-DZH**) and its conjugated base (***t*-DZ**). Both molecules have shown photochemical and chemical kinetics sensitive to the microenvironmental features. Three media have been selected in this work: they are (1) pure acetonitrile; (2) water and acetonitrile mixed in a 1/1 volume ratio, and (3) the micellar solution of zwitterionic surfactant SB3-14 dissolved in water at the concentration of 0.1 M. ***t*-DZH** exhibited the most significant photochemical quantum yield ($\Phi_{t\to c}=0.20)$ and ***c*-DZH,** the fastest thermal *cis*-to-*trans* reaction ($k_{\Delta }^{H}=\frac{1}{\tau_{LS}^{H}}=0.02\;s^{-1})$ in acetonitrile. The tight microenvironment generated by the SB3-14 micelles determines the lowest quantum yield for the trans-to-cis photochemical reaction ($\Phi_{t\to c}=0.07)$ and the slowest *cis*-to-*trans* thermal reaction ($k_{\Delta }^{H}=\frac{1}{\tau_{LS}^{H}}=0.0039\;s^{-1})$. The deprotonation of ***t*-DZH** provokes dramatic changes in its photochromic properties. The conjugated base ***t*-DZ** has shown larger photochemical quantum yields than ***t*-DZH** in the three media. In particular, the best medium to promote the photochemical *trans*-to-*cis* transformation has been the H_2_O/CH_3_CN mixture ($\Phi_{t\to c}=0.35)$, whereas the worst has been again the aqueous solution of SB3-14 micelles ($\Phi_{t\to c}=0.12)$. The absence of a proton makes ***c*-DZ** extremely lazy in isomerizing to ***t*-DZ** thermally. Nevertheless, the fastest recovery has been detected in the H_2_O/CH_3_CN mixture (it took almost 10 h), whereas the slowest one in the micellar solution (it took nearly 23 days).

**Figure 9:**
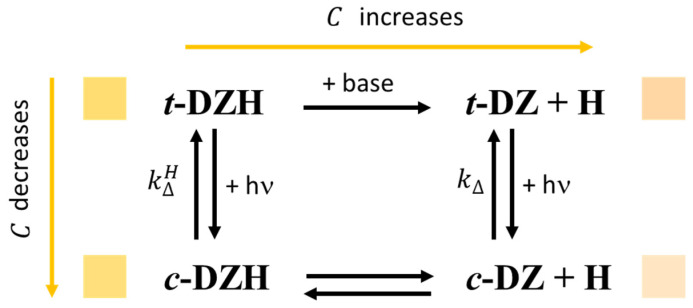
Schematic representation of the photochromic properties of ***t*-DZH** and its conjugated base, ***t*-DZ**. The colours shown next to each molecule refer to those determined in the aqueous micellar solution of SB3-14 with [***t*-DZH**] $\approx 4\times 10^{-5}\;M$.

The experimental results and their interpretation through cutting-edge algorithms, such as fuzzy entropy and colourability, demonstrate that (E)-3,4,6-trichloro-2-(*p*-diazenil)-phenol is a versatile compound to be used as a molecular probe of different micro-environments. Since its solutions are coloured, it can communicate directly to the naked eye of humans. In this work, we have shown that its colourability (defined according to the well known Shannon’s formula proposed to determine the amount of information sent in any message) decreases under UV irradiation from the *trans* to the *cis* isomer. In contrast, it grows from ***t*-DZH** to its deprotonated form, ***t*-DZ** (see Figure 9).

Finally, the versatile photochromic properties of (E)-3,4,6-trichloro-2-(*p*-diazenil)-phenol and its photoacid behaviour make it appropriate for implementing neural surrogates in the phasic excitable regime and a promising candidate for implementing photochromic oscillators, as mentioned in the introduction. ***t*-DZH** can interplay with other photochromic compounds not only through light but also through photo-induced proton exchange. It is worth pursuing the implementation of photochromic oscillators because they promise to be a breakthrough in neuromorphic engineering.

## Figures and Tables

**Table 1:** Energies, wavelengths, oscillator strengths (*f*), and characters in terms of molecular orbitals of the lowest electronic transitions for ***t*-DZH** and ***t*-DZ** (H and L stand for HOMO and LUMO). The simulated absorption spectra are reported in Figure S4 of the Supplementary Materials.

***t*-DZH**			
Transitions	E (eV)/λ (nm)	*f*	Character
S_0_→S_1_	2.47/503	0.00	99% (H-1→L)
S_0_→S_2_	3.01/412	0.30	91% (H→L); 9% (H-2→L)
S_0_→S_3_	3.41/364	0.62	91% (H-2→L); 9% (H→L)
S_0_→S_4_	3.73/332	0.03	97% (H-3→L)
***t*-DZ**			
Transitions	E (eV)/λ (nm)	*f*	Character
S_0_→S_1_	2.25/550	0.00	99% (H-1→L)
S_0_→S_2_	2.51/494	0.47	99% (H→L)
S_0_→S_3_	3.30/375	0.00	99% (H-3→L)
S_0_→S_4_	3.59/345	0.37	97% (H-2→L)

**Table 2:** Chromaticity coordinates and respective colours generated by ***t*-DZH** and ***t*-DZ** dissolved in the three media at the spectrophotometric concentrations of $5\times 10^{-5}\;M$.

	*t*-DZH				*t*-DZ				*C* (10^−3^)
	*x*	*y*	*z*	Colour	*x*	*y*	*z*	Colour	
CH_3_CN	0.339	0.365	0.296	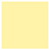	0.368	0.358	0.274	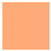	8
H_2_O/CH_3_CN = 1/1	0.336	0.359	0.305	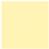	0.349	0.355	0.296	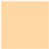	1.5
SB3-14 (0.1 M)	0.343	0.369	0.288	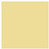	0.364	0.368	0.268	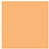	6.9

**Table 3:** Photochemical quantum yields $\Phi_{t\to c}$ for ***t*-DZH** in the three media. The relative uncertainties in the $\Phi_{t\to c}$ values are of the order of 5%.

***t*-DZH**	${\left[\mathrm{SB3-14}\right]}_{\mathrm{aq}}=0.1\;M$	$\frac{H_{2}O}{CH_{3}CN}=1/1$	$CH_{3}CN$
$\Phi_{t\to c}$	0.07	0.08	0.20

**Table 4:** Lifetimes ($\tau_{LS}^{H})$ of ***c*-DZH** determined in the three media by fitting the thermal isomerization kinetics of Figure 4A through the function of Equation (12) and the least-squares method. Normalized fuzzy entropy $H_{nor}$ for ***c*-DZH** in the three media, determined by using the output of MEM.

***t*-DZH**	${\left[\mathrm{SB3-14}\right]}_{\mathrm{aq}}=0.1\;M$	$\frac{H_{2}O}{CH_{3}CN}=1/1$	$CH_{3}CN$
$\tau_{LS}^{H}$(s)	259	137	50
$H_{\mathrm{nor}}$	0.67	0.69	0.62

**Table 5:** Photochemical quantum yields $\Phi_{t\to c}$ for ***t*-DZ** in the three media. The relative uncertainties in the $\Phi_{t\to c}$ values are of the order of 5%.

***t*-DZ**	${\left[\mathrm{SB3-14}\right]}_{\mathrm{aq}}=0.1\;M$	$\frac{CH_{3}CN}{H_{2}O}=1/1$	$CH_{3}CN$
$\Phi_{t\to c}$	0.12	0.35	0.25

**Table 6:** Lifetimes ($\tau_{LS})$ of ***c*-DZ** determined in the three media by fitting the thermal isomerization kinetics of Figure 7 through the function of Equation (11) and the least-squares method.

***t*-DZH**	${\left[\mathrm{SB3-14}\right]}_{\mathrm{aq}}=0.1\;M$	$\frac{CH_{3}CN}{H_{2}O}=1/1$	$CH_{3}CN$
$\tau_{LS}$(s)	1,960,000 ± 350,000	35,500 ± 400	41,000 ± 3000

## Data Availability

All the data are available into the Manuscript and the Supplementary Materials.

## Supplementary Materials

The following supporting information can be downloaded at: https://www.mdpi.com/article/10.3390/molecules28031183/s1, Figure S1: ***t*-DZH** spectral evolution induced by the addition of DBU in CH_3_CN; Figure S2: ***t*-DZH** spectral evolution induced by the addition of NaOH in SB3-14; Figure S3: ***t*-DZH** spectral evolution induced by the addition of NaOH in a mixture of water/acetonitrile = 1/1; Figure S4: Simulated absorption spectra of ***t*-DZH** and ***t*-DZ** in acetonitrile; Figure S5: Transmittance spectra of ***t*-DZH** and ***t*-DZ** in acetonitrile; Figure S6: Transmittance spectra of ***t*-DZH** and ***t*-DZ** in acetonitrile/water = 1/1; Figure S7: Transmittance spectra of ***t*-DZH** and ***t*-DZ** in [SB3-14]_aq_ = 0.1 M; Figure S8: Transmittance spectrum of the UG1 filter; Figure S9: Absorption spectra for the Uncolored an colored forms of the actinometer PHT; Figure S10: Spectral evolution for ***t*-DZH** under UV irradiation in H_2_O/CH_3_CN = 1/1; Figure S11: Spectral evolution for ***t*-DZH** under UV irradiation in CH_3_CN; Figure S12: Spectral evolution for ***t*-DZ** under UV irradiation in H_2_O/CH_3_CN = 1/1; Figure S13: Spectral evolution for ***t*-DZ** under UV irradiation in CH_3_CN; Figure S14: Most stable conformers for ***c*-DZH** and ***c*-DZ**; Figure S15: two conformers used to determine the strength of the intramolecular hydrogen bond. Figure S16: Spectrum of the lamp used to photo-induce ΔpH; Figure S17: ΔpH cycles for ***t*-DZH** in H_2_O/CH_3_CN = 1/1. Table S1: The CIE XYZ tristimulus and RGB values generated by ***t*-DZH** and ***t*-DZ** dissolved in the three media; Table S2: Chromaticity coordinates and respective colours generated by ***t*-DZH** before irradiation and at the photo-stationary state when dissolved in SB3-14 (0.1 M); Table S3: Chromaticity coordinates and respective colours generated by ***t*-DZH** before irradiation and at the photo-stationary state when dissolved in H_2_O/CH_3_CN = 1/1; Table S4: Chromaticity coordinates and respective colours generated by ***t*-DZH** before irradiation and at the photo-stationary state when dissolved in CH_3_CN; Table S5: Chromaticity coordinates and respective colours generated by ***t*-DZ** before irradiation and at the photo-stationary state when dissolved in SB3-14 (0.1 M); Table S6: Chromaticity coordinates and respective colours generated by ***t*-DZ** before irradiation and at the photo-stationary state when dissolved in H_2_O/CH_3_CN = 1/1; Table S7: Chromaticity coordinates and respective colours generated by ***t*-DZ** before irradiation and at the photo-stationary state when dissolved in CH_3_CN; Table S8: Kinetic constants for the photo-induced *trans*-to-*cis* isomerization for both ***t*-DZH** and ***t*-DZ**. Table S9: Optimized distances for some bonds of ***c*-DZH** and ***c*-DZ** conformers

## Sample Availability

Samples of the compounds (E)-3,4,6-trichloro-2-(*p*-diazenil)-phenol (***t*-DZH**) are available from the authors.
