# Supplementary material for: The Versatile Photo-Thermal Behaviour of a 2-Hydroxyazobenzene
Source: Molecules. 2023 Jan 25;28(3):1183. doi: 10.3390/molecules28031183 (PMC9920310; doi:10.3390/molecules28031183)
Supplement: Supplementary file 1 [file molecules-28-01183-s001.zip › molecules-2157817-supplementary.pdf]

SUPPLEMENTARY MATERIALS

The versatile photo-thermal behavior of a 2-Hydroxyazobenzene

Pier Luigi Gentili [1,*], Antonio Capaccioni [1], Raimondo Germani [1] and Simona Fantacci [2]

[1]Department of Chemistry, Biology and Biotechnology, Università degli Studi di Perugia, 06123 Perugia , Italy

[2]Istituto CNR di Scienze e Tecnologie Chimiche "Giulio Natta" (CNR-SCITEC), 06123 Perugia , Italy

*Correspondence: pierluigi.gentili@unipg.it; Tel.: +39 0755855573

Index

Paragraph	Title	Page
**1**	**Transformation of *t*-DZH into *t*-DZ after the addition of a strong base**	3
	Figure S1	3
	Figure S2	3
	Figure S3	4
**2**	**Simulated Absorption spectra**	4
	Figure S4	4
**3**	**Transmittance spectra of *t*-DZH and *t*-DZ and their colour coordinates values in the three media**	5
	Figure S5	5
	Figure S6	5
	Figure S7	6
	Table S1	6
**4**	**Photochemical experiments**	7
	Figure S8: UG1 Filter	7
	Figure S9: Absorption spectra of the actinometer	7
4.1	***Trans-to-cis photoisomerization for t-DZH in SB3-14***	8
	Table S2	8
4.2	***Trans-to-cis photoisomerization for t-DZH in H_2O/CH_3CN=1/1***	8
	Figure S10	8

	Table S3	8
4.3	***Trans-to-cis photoisomerization for t-DZH in CH_3CN***	9
	Figure S11	9
	Table S4	9
4.4	***Trans-to-cis photoisomerization for t-DZ in SB3-14***	9
	Table S5	9
4.5	***Trans-to-cis photoisomerization for t-DZ in H_2O/CH_3CN=1/1***	10
	Figure S12	10
	Table S6	10
4.6	***Trans-to-cis photoisomerization for t-DZ in CH3CN***	11
	Figure S13	11
	Table S7	11
	Table S8: kinetic constants for the photochemical reactions	12
**5**	**Optimized molecular structures through DFT calculations**	12
	Figure S14	12
	Table S9	13
	Atomic coordinates of the *trans* and *cis* isomers	13
	Figure S15	16
**6**	**Photo-induce ΔpH**	17
	Figure S16	17
	Figure S17	18

1. **Transformation of *t*-DZH into *t*-DZ after the addition of a strong base.**

***t*-DZH** at a concentration of 5×10^{-5}M in acetonitrile was completely deprotonated after successive addition of the strong organic base 1,8-diazabicyclo[5.4.0]undec-7-ene (DBU). The spectral evolution is reported in Figure S1. The final spectrum was recorded for a concentration of DBU 11 times larger than that of ***t*-DZH.**

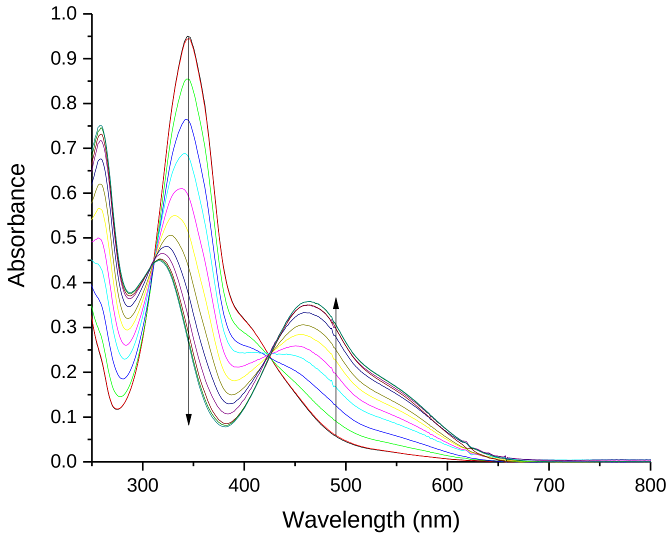

Figure S1. Spectral evolution of a ***t*-DZH** solution in acetonitrile after successive addition of DBU. The final spectrum was recorded for a ratio $\left(\frac{[DBU]}{[\boldsymbol{t-DZH}]}\right) = 11$.

***t*-DZH** at a concentration of 5×10^{-5}M in an aqueous solution of SB3-14 at 0.1 M was completely deprotonated after successive addition of NaOH. The spectral evolution is shown in Figure S2. The final spectrum was recorded for a concentration of NaOH 36 times larger than that of ***t*-DZH.**

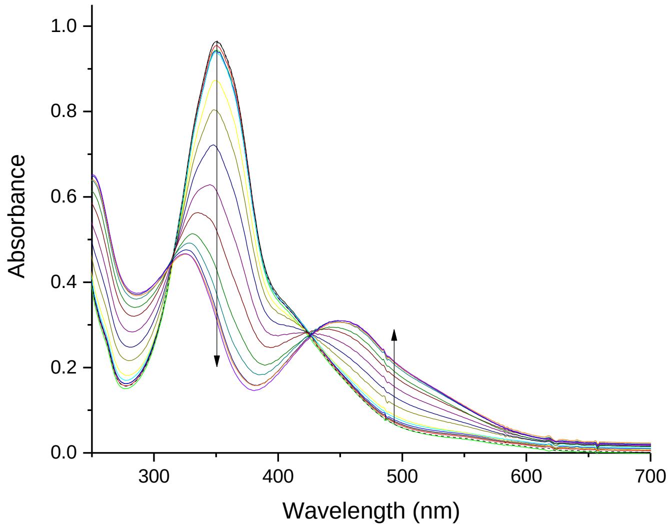

Figure S2. Spectral evolution of a ***t*-DZH** solution in $[SB3\text{-}14]_{aq}$=0.1 M after successive addition of NaOH. The final spectrum was recorded for a ratio $\left(\frac{[NaOH]}{[\boldsymbol{t-DZH}]}\right) = 36$.

***t*-DZH** at a concentration of 5×10^{-5}M in a mixture of water/acetonitrile=1/1 was completely deprotonated after a few additions of a concentrated solution of NaOH. The spectral evolution is reported in Figure S3. The final spectrum was recorded for a concentration of NaOH 12 times larger than that of ***t*-DZH.**

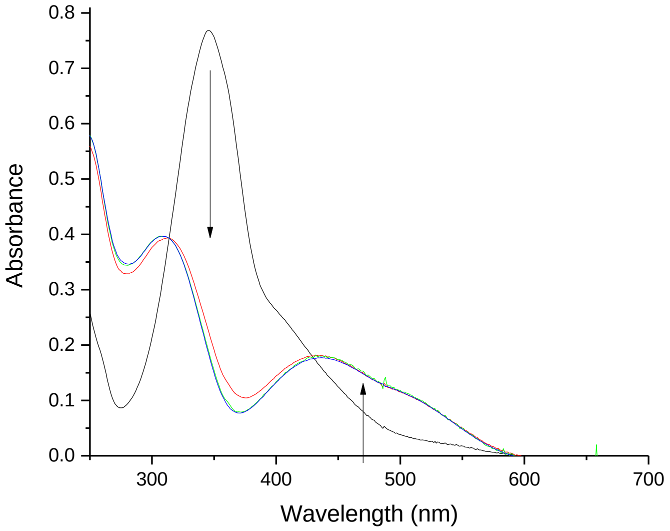

Figure S3. Spectral evolution of a ***t*-DZH** solution in a mixture of water/acetonitrile=1/1 after a few additions of a concentrated solution of NaOH. The final spectrum was recorded for a ratio $\left(\frac{[NaOH]}{[\boldsymbol{t-DZH}]}\right) = 12$.

2. Quantum-mechanical simulations

Simulated absorption spectra of ***t*-DZH** and ***t*-DZ.**

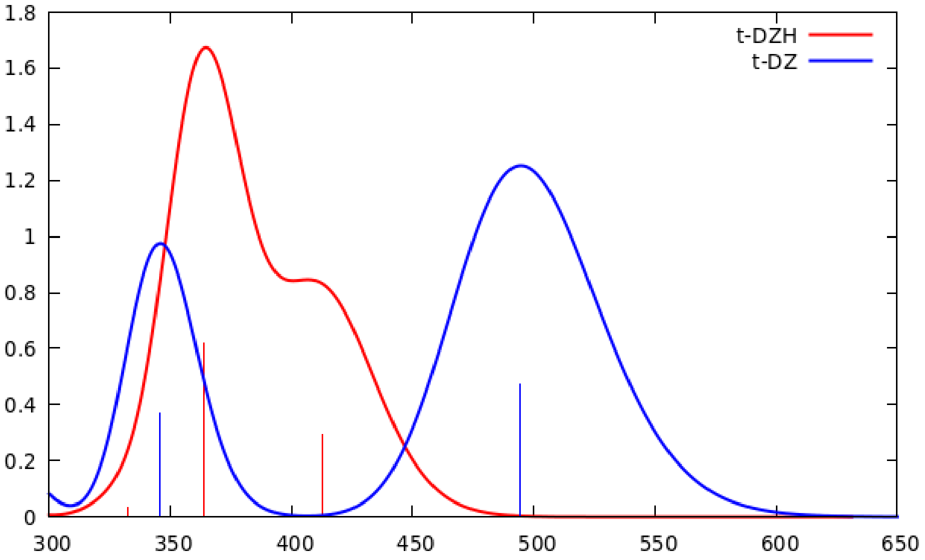

Figure S4. Simulated absorption spectra of ***t*-DZH** (red line) and ***t*-DZ** (blue line).

3. **Transmittance spectra of *t*-DZH and *t*-DZ and their colour coordinates values in the three media**

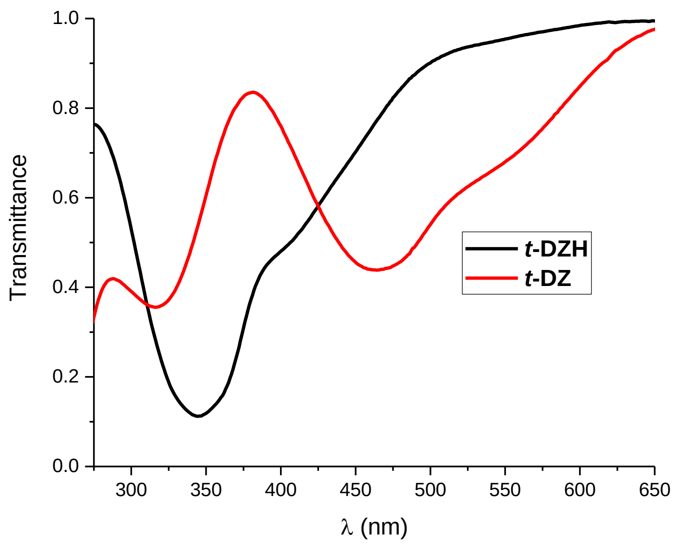

Figure S5. Transmittance spectra of ***t*-DZH** and ***t*-DZ** in acetonitrile.

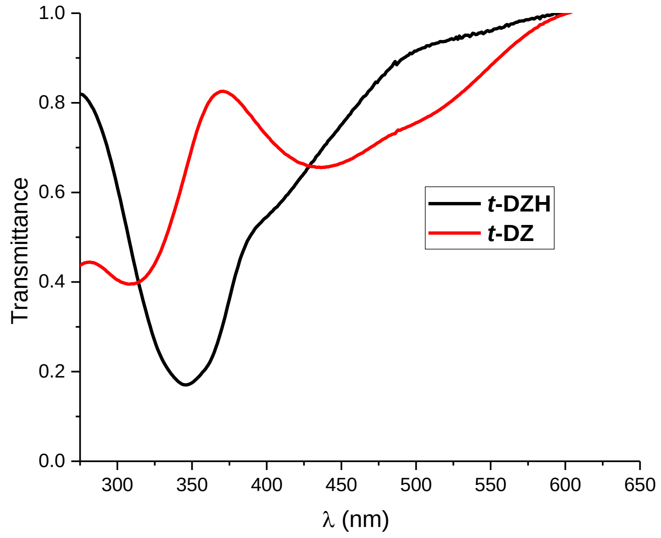

Figure S6. Transmittance spectra of ***t*-DZH** and ***t*-DZ** in water/acetonitrile=1/1.

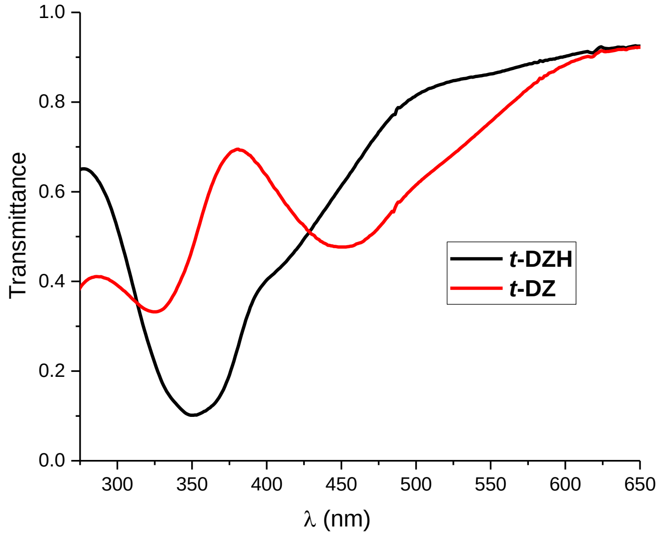

Figure S7. Transmittance spectra of ***t*-DZH** and ***t*-DZ** in [SB3-14]$_{aq}$=0.1 M

Table S1. The CIE XYZ tristimulus and RGB values generated by ***t*-DZH** and ***t*-DZ** dissolved in the three media at the spectrophotometric concentrations of 5×10^{-5}M.

Medium	***t*-DZH**						***t*-DZ**					
	X	Y	Z	R	G	B	X	Y	Z	R	G	B
CH_3CN	0.873	0.940	0.763	255	242	169	0.713	0.694	0.530	250	161	117
H_2O/CH_3CN=1/1	0.895	0.955	0.811	255	244	182	0.862	0.879	0.732	255	215	164
SB3-14 (0.1 M)	0.794	0.854	0.668	236	219	147	0.741	0.751	0.546	249	182	119

4. Photochemical experiments.

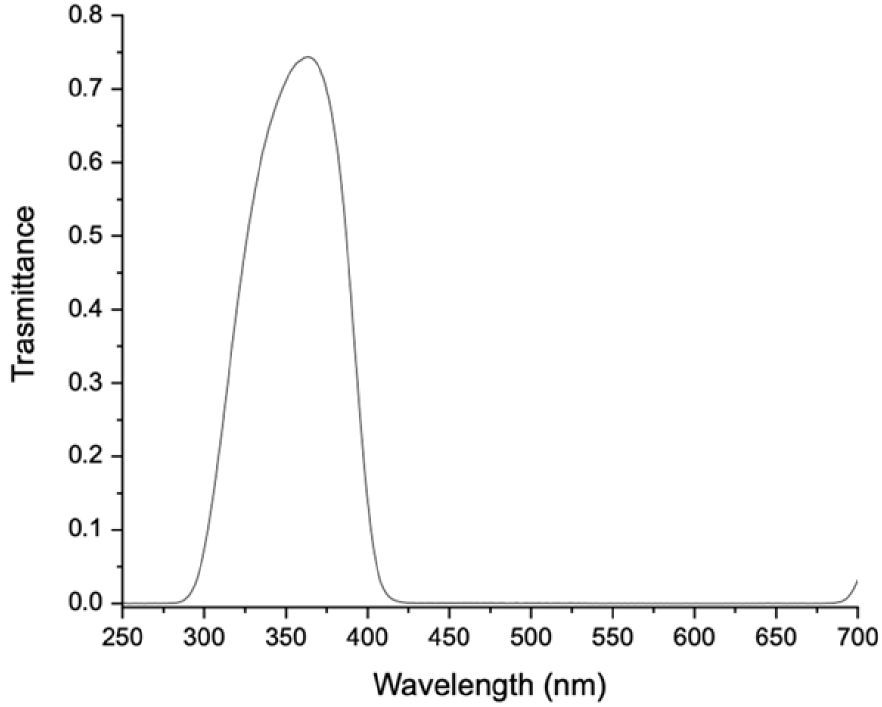

Figure S8. Transmittance spectrum of the UG1 filter.

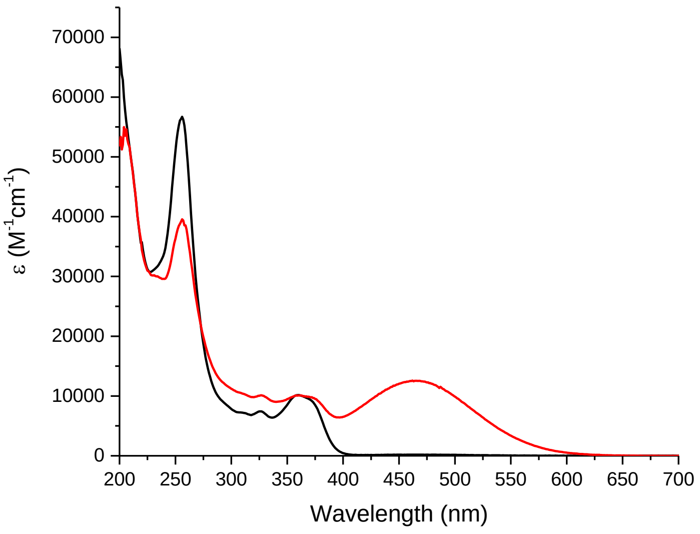

Figure S9. Absorption spectra of the uncolored (black trace) and colored (red trace) forms of the photochromic PHT used as an actinometer.

4.1 Trans-to-cis photoisomerization for t-DZH in SB3-14

Table S2. Chromaticity coordinates and respective colours generated by ***t*-DZH** before irradiation and at the photo-stationary state when dissolved in the micellar solution of SB3-14 (0.1 M) and at the spectrophotometric concentration of 4.2×10^{-5}M.

	Initial Spectrum				*Ph.-station. State Spectrum*				C (10^{-3})
	x	y	z	colour	x	y	z	colour	
SB3-14 (0.1 M)	0.339	0.363	0.297		0.335	0.357	0.308		-2.3

4.2 Trans-to-cis photoisomerization for t-DZH in H_2O/CH_3CN=1/1

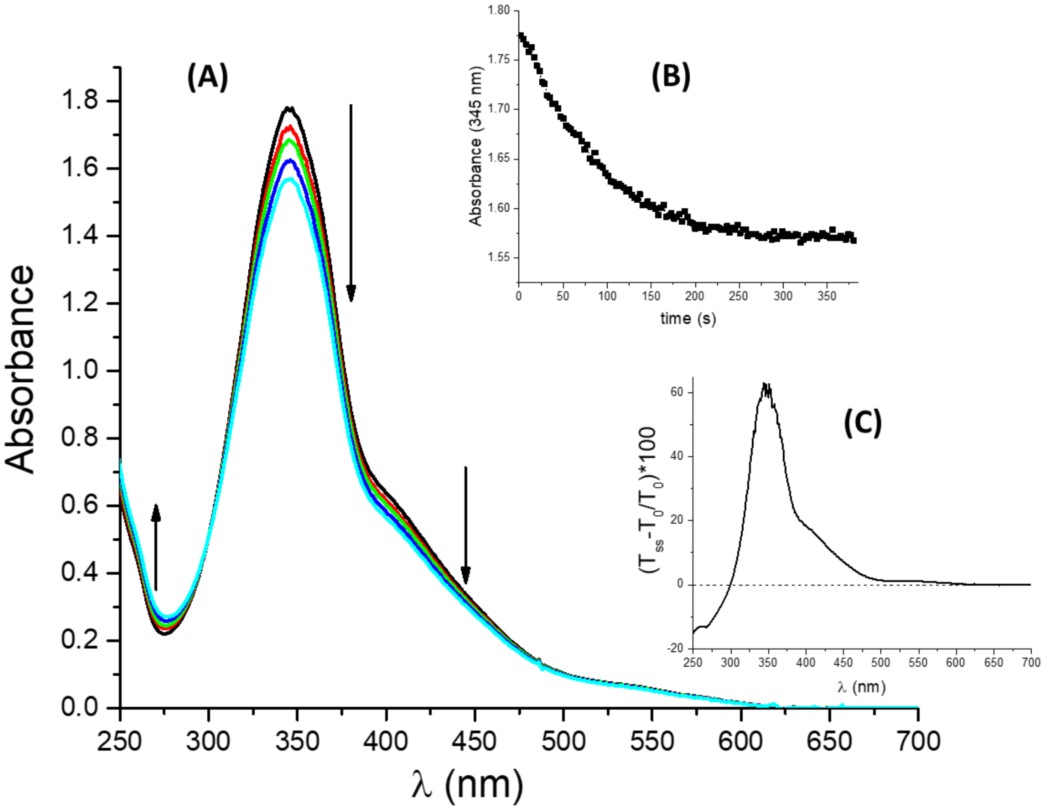

Figure S10. Spectral modifications (A) induced by a steady UV irradiation at $\lambda_{irr} = (363 \pm 40)nm$ for [***t*-DZH**]=1.0×10^{-4}M in H_2O/ CH_3CN=1/1: the arrows indicate the trends of absorbance in the different spectral regions (the black and cyan traces are the initial and final spectra, recorded before irradiation and at the photo-stationary state, respectively). Graph B shows how the absorbance at 345 nm changes upon UV irradiation. Graph C reports the trend of the relative percentage transmittance change, defined in equation (3), as the function of wavelength.

Table S3. Chromaticity coordinates and respective colours generated by ***t*-DZH** before irradiation and at the photo-stationary state when dissolved in H_2O/ CH_3CN=1/1 at the spectrophotometric concentration of 1.0×10^{-4}M.

	Initial Spectrum				*Ph.-station. State Spectrum*				C (10^{-3})
	x	y	z	colour	x	y	z	colour	
CH_3CN	0.364	0.390	0.246		0.360	0.385	0.256		-5.8

4.3 Trans-to-cis photoisomerization for t-DZH in CH_3CN

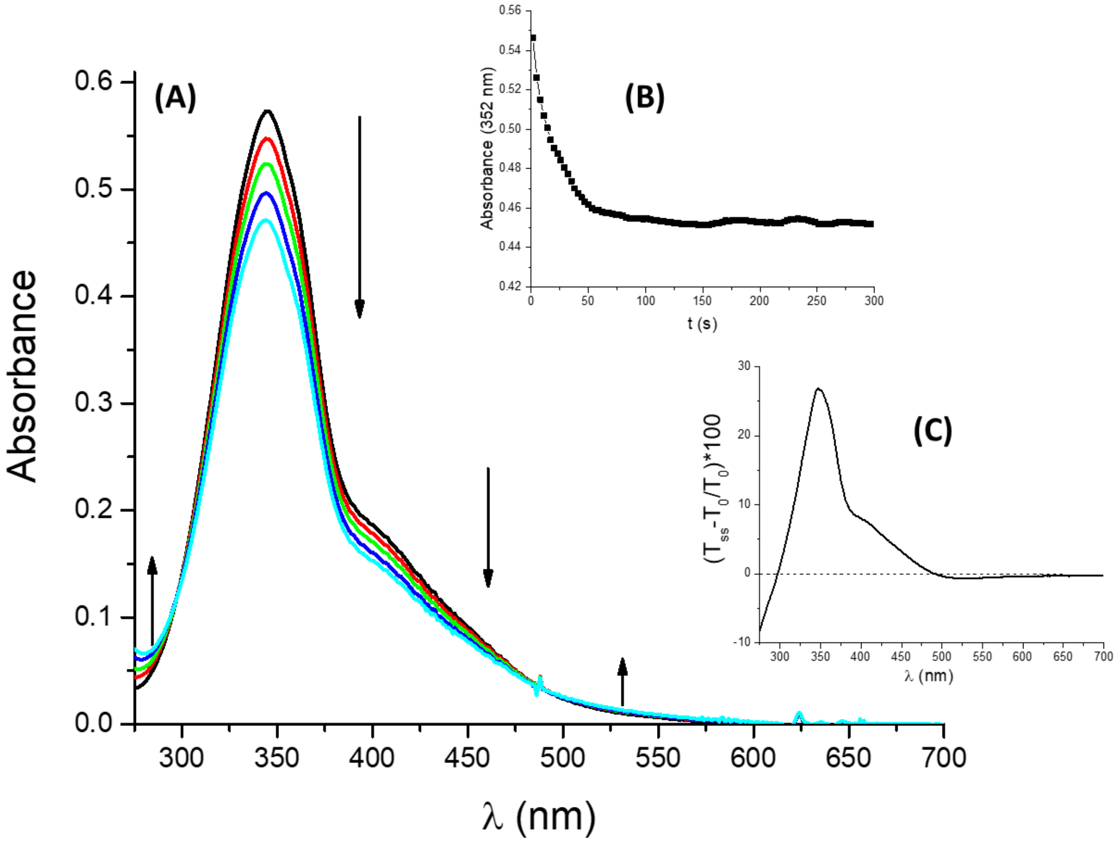

Figure S11. Spectral modifications (A) induced by a steady UV irradiation at $\lambda_{irr} = (363 \pm 40)nm$ for [***t*-DZH**]=3.2×10^{-5}M in CH_3CN: the arrows indicate the trends of absorbance in the different spectral regions (the black and cyan traces are the initial and final spectra, recorded before irradiation and at the photo-stationary state, respectively). Graph B shows how the absorbance at 352 nm changes upon UV irradiation. Graph C reports the trend of the relative percentage transmittance change, defined in equation (3), as the function of wavelength.

Table S4. Chromaticity coordinates and respective colours generated by ***t*-DZH** before irradiation and at the photo-stationary state when dissolved in acetonitrile at the spectrophotometric concentration of 3.2×10^{-5}M.

	Initial Spectrum				*Steady-state Spectrum*				C (10^{-3})
	x	y	z	colour	x	y	z	colour	
CH_3CN	0.329	0.353	0.318		0.323	0.356	0.321		0.3

4.4 Trans-to-cis photoisomerization for t-DZ in SB3-14

Table S5. Chromaticity coordinates and respective colours generated by ***t*-DZ** before irradiation and at the photo-stationary state when dissolved in the micellar solution of SB3-14 (0.1 M) and at the spectrophotometric concentration of 4.8×10^{-5}M.

	Initial Spectrum				*Ph.-station. State Spectrum*				C (10^{-3})
	x	y	z	colour	x	y	z	colour	
SB3-14 (0.1 M)	0.361	0.368	0.271		0.344	0.354	0.302		-9.8

4.5 Trans-to-cis photoisomerization for t-DZ in H_2O/CH_3CN=1/1

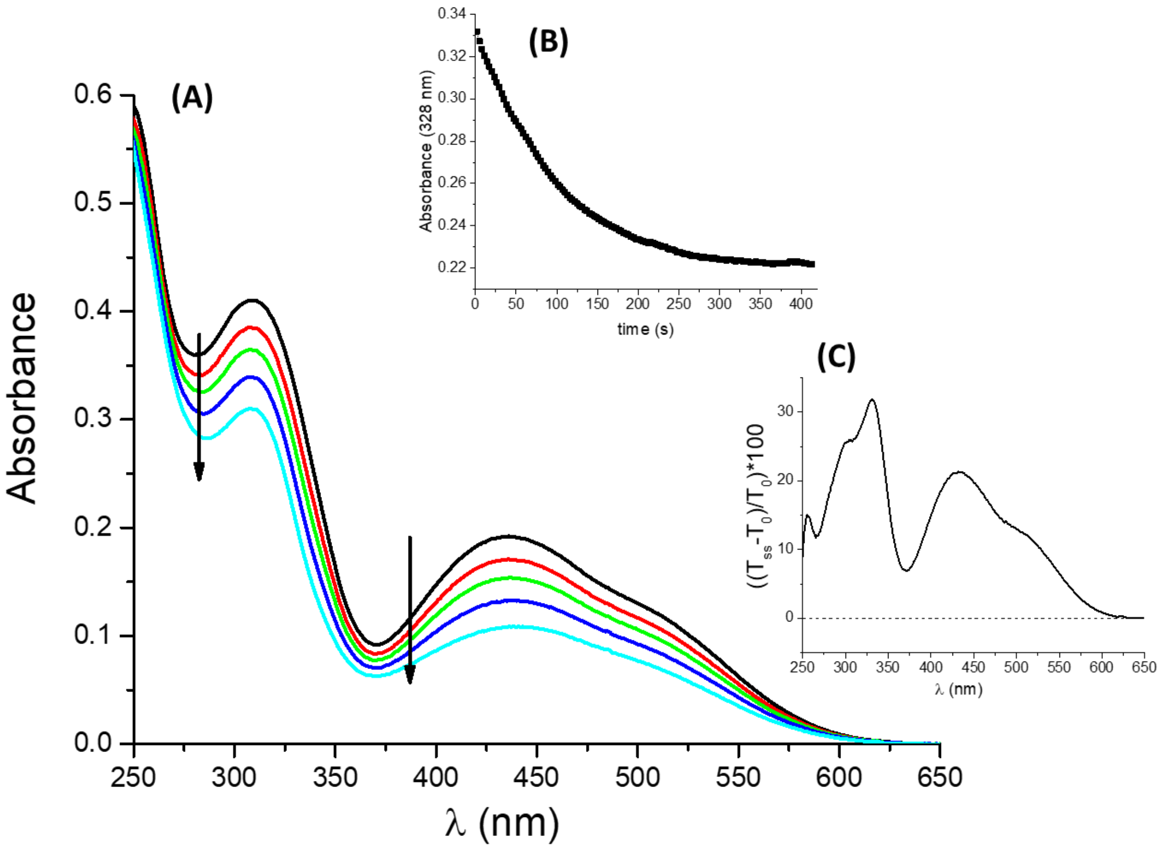

Figure S12. Spectral modifications (A) induced by a steady UV irradiation at $\lambda_{irr} = (363 \pm 40)nm$ for [***t*-DZ**]=5 × 10^{-5}M in H_2O/CH_3CN=1/1: the arrows indicate the trends of absorbance in the different spectral regions (the black and cyan traces are the initial and final spectra, recorded before irradiation and at the photo-stationary state, respectively). Graph B shows how the absorbance at 328 nm changes upon UV irradiation. Graph C reports the trend of the relative percentage transmittance change, defined in equation (3), as the function of wavelength.

Table S6. Chromaticity coordinates and respective colours generated by ***t*-DZ** before irradiation and at the photo-stationary state when dissolved in H_2O/ CH_3CN=1/1 at the spectrophotometric concentration of 5.0 × 10^{-5}M.

	Initial Spectrum				*Ph.-station. State Spectrum*				C (10^{-3})
	x	y	z	colour	x	y	z	colour	
CH_3CN	0.349	0.355	0.296		0.334	0.345	0.321		-4.0

4.6 Trans-to-cis photoisomerization for t-DZ in CH_3CN

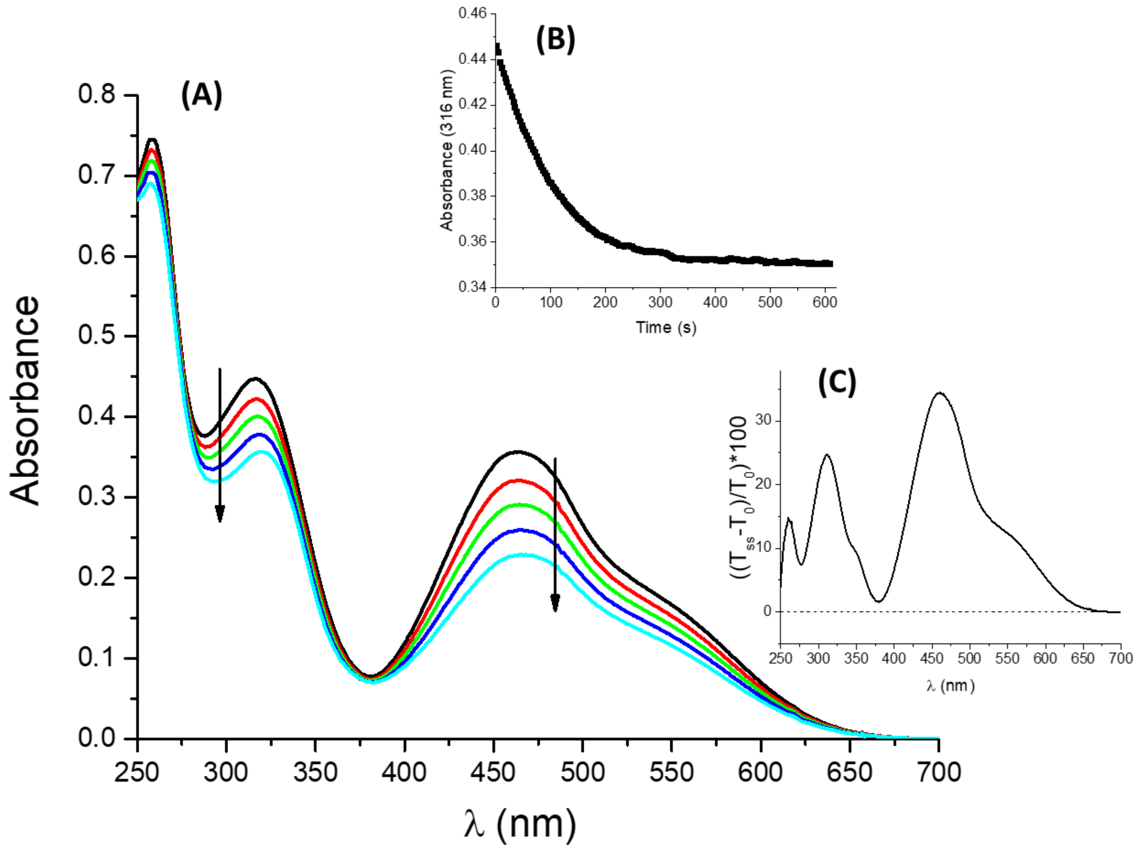

Figure S13. Spectral modifications (A) induced by a steady UV irradiation at $\lambda_{irr} = (363 \pm 40)nm$ for [***t*-DZ**]=5 × 10^{-5}M in CH_3CN: the arrows indicate the trends of absorbance in the different spectral regions (the black and cyan traces are the initial and final spectra, recorded before irradiation and at the photo-stationary state, respectively). Graph B shows how the absorbance at 316 nm changes upon UV irradiation. Graph C reports the trend of the relative percentage transmittance change, defined in equation (3), as the function of wavelength.

Table S7. Chromaticity coordinates and respective colours generated by ***t*-DZ** before irradiation and at the photo-stationary state when dissolved in CH_3CN at the spectrophotometric concentration of 5.0 × 10^{-5}M.

	Initial Spectrum				*Ph.-station. State Spectrum*				C (10^{-3})
	x	y	z	colour	x	y	z	colour	
CH_3CN	0.368	0.358	0.274		0.349	0.347	0.304		-9.1

Table S8. Kinetic constant values k(s^{-1}) for the photochemical conversion of trans to cis determined by fitting the ΔA changes vs. time through the following function: $\Delta A = A_{\infty} + A' e^{-\frac{t}{k}}$.

	SB3-14	H_2O/CH_3CN=1/1	CH_3CN
***t*-DZH**	0.018	0.012	0.045
***t*-DZ**	0.0077	0.010	0.010

5. Optimized molecular structures through DFT calculations

In the attempt to determine the most stable structure for ***c*-DZH**, distinct conformers with slightly different potential energies (within 1 kcal/mol) have been found. Three conformers are shown in Figure S14, starting from the most stable one (***c*-DZH_0**). Its energy has been fixed as the reference for determining the relative energies of the other two conformers (***c*-DZH_1** and ***c*-DZH_2**).

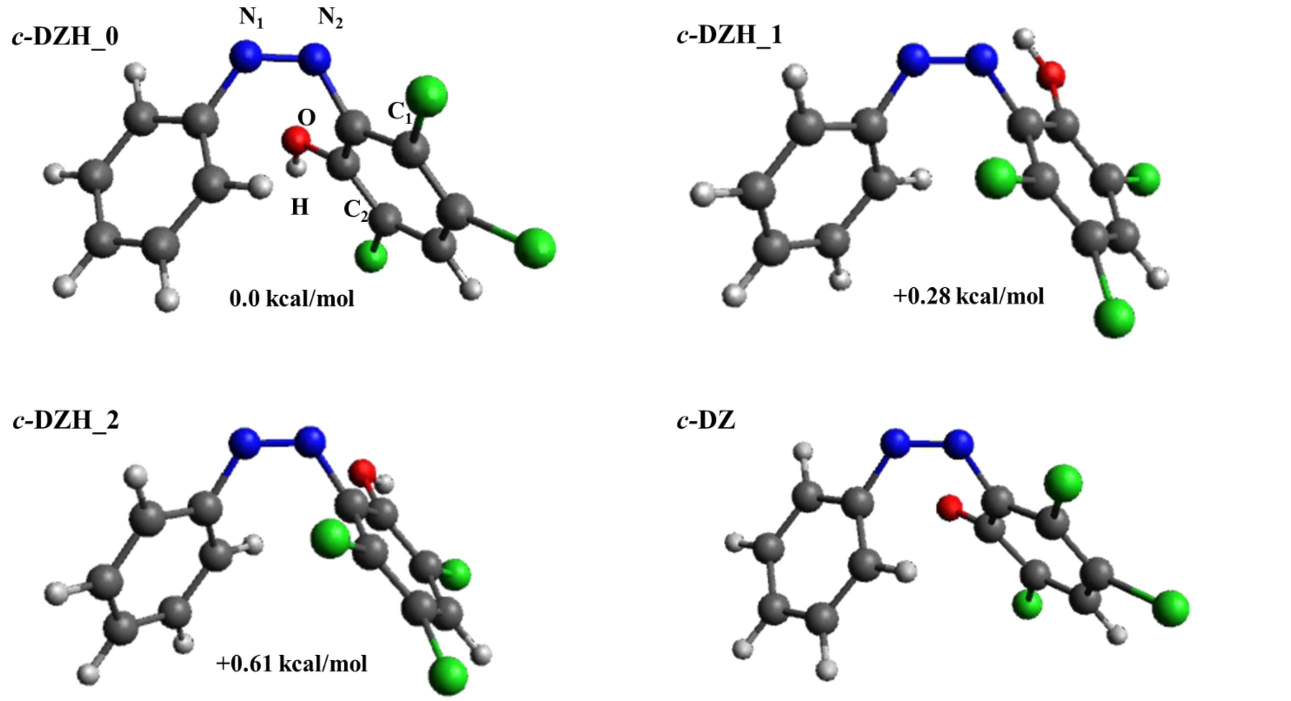

Figure S14. Structures of three among the most stable conformers of ***c*-DZH**: ***c*-DZH_0**, which is the most stable, ***c*-DZH_1**, and ***c*-DZH_2**. C-DZ is the most stable conformer of its coniugated base.

Table S9 reports the optimized distances of some bonds that play a relevant role in the photochromism of the *o*-hydroxyazobenzene.

Table S9. Optimized distances for some bonds of ***c*-DZH** and ***c*-DZ** conformers depicted in Figure S14.

Optimized bond distances (Å)	***c*-DZH_0**	***c*-DZH_1**	***c*-DZH_2**	***c*-DZ**
N_1N_2	1.244	1.248	1.241	1.252
N_2C_1	1.439	1.434	1.444	1.419
C_1C_2	1.403	1.406	1.400	1.457
C_2O	1.349	1.350	1.352	1.260
OH	0.969	0.971	0.969	_-

Atomic coordinates of the optimized molecular structures:

t-DZH

C	-0.127878	0.000015	0.301430
C	0.093534	-0.000001	1.699933
C	1.404878	-0.000017	2.181430
C	2.492630	-0.000018	1.310309
C	2.286881	-0.000002	-0.057454
C	0.991386	0.000014	-0.576643
Cl	-1.217395	-0.000001	2.842306
Cl	1.751093	-0.000037	3.899227
H	3.498307	-0.000031	1.707185
Cl	3.653430	-0.000003	-1.147081
O	0.808510	0.000029	-1.905594
N	-1.323061	0.000034	-0.435777
H	-0.159497	0.000039	-2.042469
N	-2.428480	0.000037	0.157051
C	-3.574850	0.000016	-0.671755
C	-4.795690	0.000019	0.017294
C	-5.996801	0.000000	-0.686035
C	-5.983845	-0.000021	-2.080193
C	-4.765940	-0.000023	-2.770787
C	-3.563980	-0.000005	-2.077089
H	-4.780972	0.000035	1.100639
H	-6.937989	0.000002	-0.149251
H	-6.916862	-0.000035	-2.631789
H	-4.760281	-0.000039	-3.854644
H	-2.624816	-0.000006	-2.612815

t-DZ

C	-0.120266	0.000008	0.337134
C	0.112346	0.000018	1.724003
C	1.432996	0.000010	2.196027
C	2.503904	-0.000008	1.305127
C	2.268673	-0.000018	-0.069821
C	0.952943	-0.000010	-0.546125
N	-0.895275	0.000037	2.724392
N	-2.076945	0.000042	2.271385
C	-3.170738	0.000060	3.118713

C	-3.192729	0.000065	4.545973
C	-4.396027	0.000088	5.235117
C	-5.618912	0.000107	4.542216
C	-5.633849	0.000101	3.175565
C	-4.438299	0.000075	2.357910
Cl	-1.720232	0.000039	5.505807
Cl	-4.483914	0.000094	6.996476
Cl	-7.188613	0.000126	2.343514
O	-4.495114	0.000069	1.107049
H	-6.544422	0.000126	5.102754
H	1.601272	0.000018	3.266968
H	3.519942	-0.000015	1.683676
H	3.099629	-0.000032	-0.766087
H	0.767204	-0.000018	-1.614831
H	-1.138546	0.000014	-0.028094

***c*-DZH_0**

E(RB3LYP) = -2026.60455698

N	-0.254546	-0.339150	0.181753
C	-0.173547	-0.223413	1.601039
C	1.100099	-0.252230	2.174916
C	1.240387	-0.242905	3.558440
C	0.109694	-0.251854	4.377077
C	-1.161799	-0.274936	3.801673
C	-1.309917	-0.258059	2.418110
H	1.967760	-0.260676	1.526387
H	2.230601	-0.241352	3.998829
H	0.218176	-0.258392	5.455182
H	-2.042369	-0.311117	4.432578
H	-2.296759	-0.289944	1.976704
N	-1.096151	0.288233	-0.500816
C	-1.851052	1.369991	0.021462
C	-3.213023	1.420974	-0.288773
C	-3.994719	2.512463	0.073251
C	-3.390859	3.598533	0.710589
C	-2.037439	3.581432	0.970091
C	-1.159735	2.488903	0.648282
Cl	-3.948047	0.022308	-1.062627
Cl	-5.724709	2.569165	-0.236226
H	-3.990375	4.452716	0.994966
Cl	-1.319330	4.978317	1.776351
O	0.082227	2.498085	0.857732

***c*-DZH_1**

Energy=-2027.06432261

C	-2.860325	-0.799000	1.207206
C	-3.297212	0.413649	0.656942
C	-4.433349	1.052930	1.165402
C	-5.080726	0.528745	2.277531
C	-4.634330	-0.665508	2.844242
C	-3.539221	-1.334994	2.294439
N	-2.724754	1.009504	-0.500350

N	-1.500430	1.029849	-0.738863
C	-0.490621	0.577527	0.172943
C	-0.246934	1.117118	1.441699
C	0.849502	0.663589	2.177647
C	1.704929	-0.297460	1.650131
C	1.489766	-0.787349	0.370197
C	0.412353	-0.339333	-0.393045
Cl	-1.280064	2.368461	2.068136
Cl	1.189397	1.292721	3.772906
Cl	2.581657	-1.973563	-0.301402
O	0.222746	-0.819088	-1.640973
H	2.546530	-0.648535	2.229919
H	-0.486647	-0.308828	-2.064709
H	-4.775274	1.966621	0.695277
H	-5.941990	1.039496	2.690295
H	-5.149223	-1.084822	3.700143
H	-3.214214	-2.279562	2.713263
H	-2.024295	-1.332708	0.778400

***c*-DZH_2**

Energy= -2027.06380682

C	1.637237	-0.221537	1.755415
C	1.335121	-0.848277	0.555224
C	0.282276	-0.412474	-0.249005
Cl	-1.136266	2.693439	1.877695
Cl	1.280270	1.641634	3.679456
H	2.459708	-0.572534	2.361860
Cl	2.277236	-2.225949	0.021807
O	-0.075378	-1.003546	-1.411373
N	-1.468602	1.194687	-0.740543
H	0.533410	-1.729483	-1.612709
N	-2.690784	1.137439	-0.528754
C	-3.248407	0.442039	0.588300
C	-4.280779	1.094234	1.267445
C	-4.886793	0.473773	2.353916
C	-4.503531	-0.815748	2.725041
C	-3.512674	-1.484859	2.005792
C	-2.874644	-0.859583	0.939372
H	-4.576767	2.086984	0.951786
H	-5.667538	0.988447	2.900539
H	-4.988984	-1.305586	3.560495
H	-3.237478	-2.498135	2.272129
H	-2.128124	-1.391141	0.366266

***c*-DZ**

E(RB3LYP) = -2026.60258112

C	-0.506725	0.688569	0.182175
C	-0.194328	1.375587	1.348424
C	0.851563	0.935624	2.165702
C	1.580176	-0.189589	1.792425
C	1.268414	-0.862636	0.624349
C	0.210471	-0.481875	-0.258745

Cl	-1.102181	2.819878	1.777953
Cl	1.287801	1.777649	3.647998
H	2.391002	-0.535026	2.419467
Cl	2.206889	-2.297245	0.200944
O	-0.131061	-1.112913	-1.305578
N	-1.466945	1.211519	-0.758623
N	-2.690316	1.156839	-0.548159
C	-3.230603	0.454270	0.581141
C	-4.198562	1.111389	1.341231
C	-4.796272	0.452466	2.411991
C	-4.463339	-0.873203	2.691349
C	-3.528800	-1.539182	1.896391
C	-2.903849	-0.880256	0.841852
H	-4.457029	2.135667	1.101571
H	-5.530364	0.969034	3.018855
H	-4.940429	-1.390276	3.515395
H	-3.285872	-2.576027	2.097213
H	-2.190347	-1.394000	0.210086

In the attempt to determine the strength of the intramolecular hydrogen bond between the hydroxylic and azo groups, the energy difference between the two conformers shown in Figure S15 has been determined through the computational methodology explained in paragraph 3.6 of the main article.

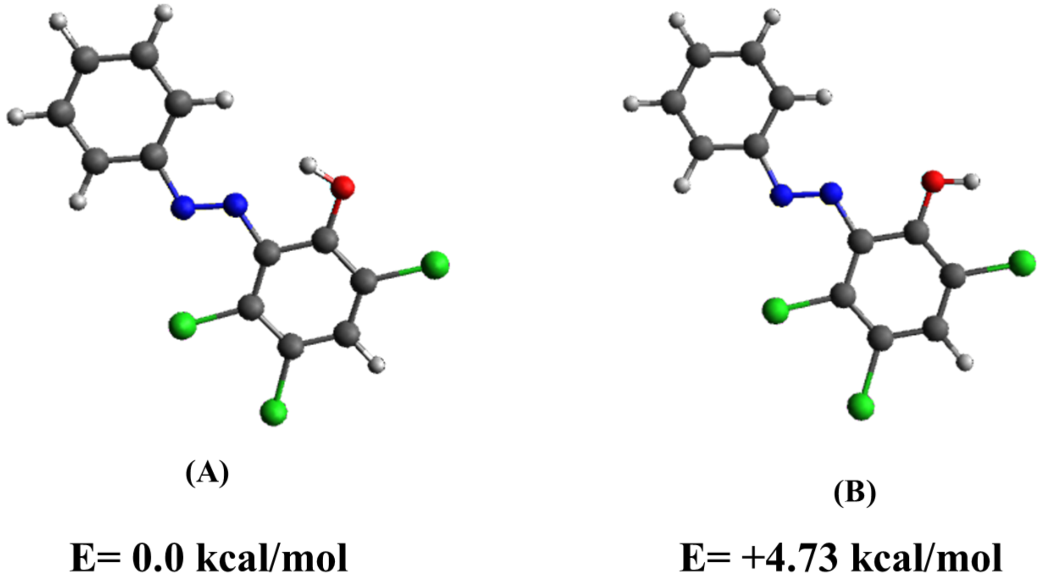

Figure S15. The two conformers (A and B) used to determine the strength of the intramolecular hydrogen bond between the hydroxylic and azo groups. The hydrogen bond is present in the conformer A on the left, whereas it is not in the conformer B shown on the right.

Molecular coordinates of the conformer B of Figure S15:

C	-3.559364	-0.000008	-2.064191
C	-3.573921	-0.000005	-0.659255
C	-4.794543	0.000002	0.027858
C	-5.994705	0.000007	-0.678494
C	-5.979474	0.000004	-2.072712
C	-4.760101	-0.000003	-2.760815

N	-2.418300	-0.000011	0.163722
N	-1.329607	-0.000014	-0.459234
C	-0.123540	-0.000006	0.266864
C	0.088550	-0.000005	1.670867
C	1.391258	0.000000	2.178818
C	2.492375	0.000006	1.329563
C	2.296185	0.000006	-0.037729
C	1.011242	0.000000	-0.590605
Cl	-1.227660	-0.000015	2.808805
Cl	1.710354	0.000000	3.902094
Cl	3.679273	0.000012	-1.117131
O	0.811827	-0.000001	-1.921793
H	3.493034	0.000011	1.738408
H	1.669034	0.000003	-2.374240
H	-4.782546	0.000004	1.111404
H	-6.937168	0.000013	-0.143655
H	-6.911770	0.000008	-2.625725
H	-4.752790	-0.000006	-3.844881
H	-2.612267	-0.000015	-2.586890

6. Photo-induce ΔpH

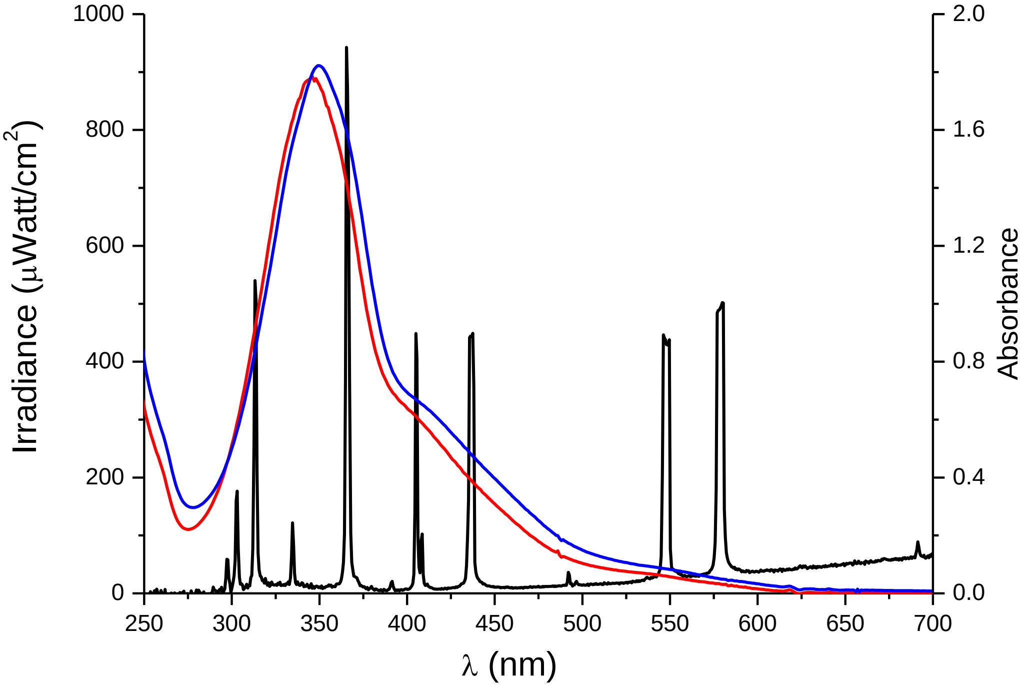

Figure S16. Irradiance and spectral profile (in black) emitted by the lamp used to photo-induce the ΔpH shown in Figure 8 of the main text. Absorption spectra of [***t*-DZH**]=1 × $10^{-4} M$ in H_2O/CH_3CN=1/1 (red spectrum) and in the micellar aqueous solution with [SB3-14]=0.1 M (blue spectrum).

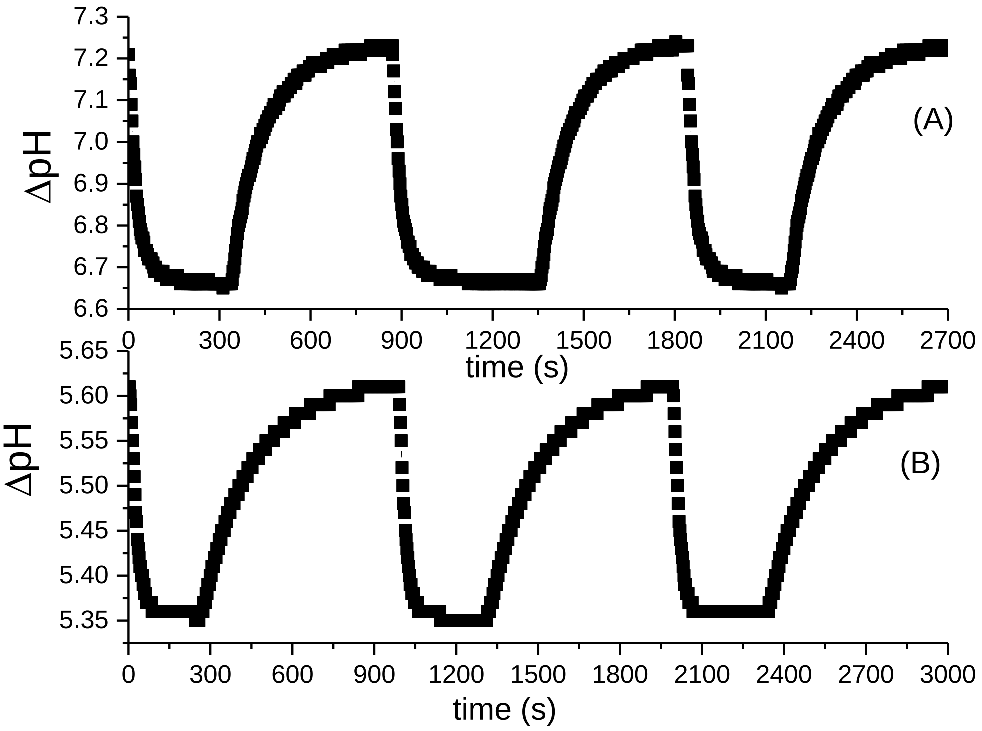

Figure S17. Three consecutive ΔpH cycles generated by the irradiation of ***t*-DZH** in H_2O/CH_3CN=1/1 (A) and SB3-14 (B).
